# On the drivers of ice nucleating particle diurnal variability in Eastern Mediterranean clouds

**DOI:** 10.1038/s41612-024-00817-9

**Published:** 2025-05-05

**Authors:** Kunfeng Gao, Franziska Vogel, Romanos Foskinis, Stergios Vratolis, Maria I. Gini, Konstantinos Granakis, Olga Zografou, Prodromos Fetfatzis, Alexandros Papayannis, Ottmar Möhler, Konstantinos Eleftheriadis, Athanasios Nenes

**Affiliations:** 1 Laboratory of Atmospheric Processes and Their Impacts, School of Architecture, Civil and Environmental Engineering, École Polytechnique Fédérale de Lausanne, Lausanne, Switzerland; 2 Institute of Meteorology and Climate Research, Karlsruhe Institute of Technology, Karlsruhe, Germany; 3 Laser Remote Sensing Unit (LRSU), Physics Department, National Technical University of Athens, Zografou, Greece; 4 Centre for the Study of Air Quality and Climate Change, Institute of Chemical Engineering Sciences, Foundation for Research and Technology Hellas, Patras, Greece; 5 ENvironmental Radioactivity & Aerosol Technology for atmospheric & Climate ImpacT Lab, INRASTES, NCSR Demokritos, Attica, Greece; 6 Present Address: Institute for Atmospheric and Climate Science, ETH Zurich, Zurich, Switzerland; 7 Present Address: Institute of Atmospheric Sciences and Climate (ISAC), National Research Council (CNR), Bologna, Italy

**Keywords:** Atmospheric chemistry, Atmospheric science

## Abstract

We report the drivers of spatiotemporal variability of ice nucleating particles (INPs) for mixed-phase orographic clouds (~−25 °C) in the Eastern Mediterranean. In the planetary boundary layer, pronounced INP diurnal periodicity is observed, which is mainly driven by biological (and to a lesser extent, dust) particles but not aerosols from biomass burning. The comparison of size-resolved and fluorescence-discriminated aerosol particle properties with INPs reveals the primary role of fluorescent bioaerosol. The presence of Saharan dust increases INPs during nighttime more than daytime, because of lower boundary layer height during nighttime which decreases the contribution of aerosols (including bioaerosols) from the boundary layer. INP diurnal periodicity is absent in the free troposphere, although levels are driven by the availability of bioaerosol and dust particles. Given the effective ice nucleation ability of bioaerosols and subsequent effects from ice multiplication at warm temperatures, the lack of such cycles in models points to important and overlooked drivers of cloud formation and precipitation in mountainous regions.

## Introduction

Atmospheric ice nucleation plays a vital role in cloud formation and cloud microphysical properties, which considerably influences regional and global precipitation, hydrological cycle^1^, atmospheric radiative forcing^2^ and the Earth’s energy balance^3,4^. For temperature (*T*) lower than −38 °C, atmospheric ice formation can occur spontaneously via homogeneous freezing^5^. However, for warmer temperatures, the initiation of cloud ice formation in most clouds necessitates ice nucleating particles (INPs) that heterogeneously freeze^6^. Considering the strong impacts that INPs can have on cloud properties, though a minor fraction of total particles^7^, INPs can bear large impacts on the hydrological cycle and the climate^8^.

The ice formation ability and the abundance of INPs depend on temperature, particle types and their degree of atmospheric aging^9^. It is well known that dust particles from desert and agricultural lands^7^, biological particles, and soil dust particles containing biological materials^9^ constitute major sources of INPs for warmer mixed-phase clouds (MPCs), along with regionally sourced black carbon and organic particles from biomass burning emissions for colder cirrus clouds^4,6^. Moreover, airmass transport^10,11^ and atmospheric aging processes^12,13^ can modulate INP concentrations and characteristics^14^. The large uncertainty in the spatiotemporal variability of INPs^7,15^ (both abundance and distribution), together with the uncertainty in subsequent cloud processes such as ice multiplication^16–18^, leads to not fully constrained effects of INPs in regional and global weather, climate, and Earth system models^4,9^. Therefore, there is a significant need to improve the predictability of INPs in models^8^.

Periodic (seasonal and diurnal) solar radiation and anthropogenic activities may induce corresponding cycles on aerosol sources^19^, hence INPs^20,21^. Previous studies reported a seasonal periodicity of INPs in different regions^22–24^; studies on INP variabilities within a day are still scarce, owing to the insufficient time resolution of offline INP spectrometers, short duration of observations, and incomplete attribution of INP sources. Diurnal variability of INPs can be an especially important driver for ice formation in orographic cloud systems, given their dynamic nature. Rosinski et al.^25^ found that INPs (at −15 and −20 °C) show concentration maxima at 06:00 and 18:00 local time of the day. Despite the short duration and low time resolution of the data (4 h), the results from Rosinski et al.^25^ suggested an INP diurnal cycle that subsequent studies supported^11,23^, albeit with limited insights on the implications. Wieder et al.^11^ observed INPs at the Weissfluhjoch mountaintop (2693 m a.sl., Alps) at Davos, Switzerland every 2 h using an offline droplet freezing assay and found a diurnal cycle of INPs showing a minimum in the morning and a peak after sunset. Night time observations, however, were missing^11^. Not all studies exhibit a diurnal cycle. Isono et al.^26^ reported no appreciable diurnal variations for INPs (at −15 °C) observed at the Manuna Loa observatory at ~3400 m above sea level (a.s.l.) (Hawaii, US) using an offline static cloud chamber to measure INPs every ~6 h. The availability of automated online INP spectrometers^27,28^ with high temporal resolution facilitates investigations on the INP diurnal periodicity. Using an automated continuous flow diffusion chamber, Brunner et al.^23^ performed year-long continuous INP observations at a high altitude observatory at Jungfraujoch (~3580 m a.s.l., Alps, Switzerland) and reported the diurnal periodicity of INPs for days only with planetary boundary layer (PBL) air mass intrusions without Saharan dust events. Detailed sources responsible for these diurnal cycles were still underexplored^23^. Temporal changes of INPs also depend on the airmass origin at the site, given that the aerosol diurnal cycles differ between the PBL and free tropospheric (FT)^29,30^ aerosols. Contrast to these studies, Wieder et al.^11^ found no diurnal cycles of INPs at Wolfgangpass (1632 m a.s.l., Alps, at Davos) which tends to reside in the PBL during the observation period, different from another in parallel observation site near Weissfluhjoch ( ~ 1.0 km higher) at the mountaintop close to the FT. Therefore, determining the atmospheric condition of the observation site is necessary for investigating the INP abundance and its diurnal periodicity.

In mountainous regions, local or regional biological particle^20^ sources in the PBL exhibiting a diurnal cycle may drive INP variability; the diurnal changes of PBLH may also regulate the availability of aerosol particles from the PBL^31^. Of particular interest is the importance of bioaerosols, since most often forested areas are in mountainous regions. If not forested, arid mountains could also be sources of dust, given that mountain flows in general can generate high velocities that are capable of lifting large amounts of coarse mode particles. Such processes are poorly described or lacking in models, thus it is critical to assess their importance for INP diurnal variability. This is because when combined with the effects of ice multiplication, these INP sources can rapidly glaciate clouds and generate heavy snowfall and extreme precipitation close to the ground during winter storms, as demonstrated by Georgakaki et al.^18^.

In this study, we present the drivers for diurnal cycles of INPs under different atmospheric conditions. The observations were collected during a field campaign, the Cloud-AerosoL InteractionS in the Helmos background TropOsphere (CALISHTO, https://calishto.panacea-ri.gr/) between October and November, 2021. CALISHTO was conducted at the Helmos Hellenic Atmospheric Aerosol and Climate Change (in short as (HAC)^2^ hereafter) station (37.9843° N, 22.1963° E, 2314 m a.s.l.) close to the summit of Mt. Helmos in the Pelloponnese. Using the PBL characteristics and INP source apportionment in companion studies^32,33^, we determine the diurnal cycle of INPs for a variety of atmospheric states represented by the relative position of the (HAC)^2^ with respect to the PBL height (PBLH), airmass origin and the effect of Saharan dust events. The distinct contributions of bioaerosol and dust to the diurnal cycle are determined for different locations in the atmospheric column.

## Results

### Determination of atmospheric conditions at (HAC)^2^

(HAC)^2^ contributes data to the Global Atmospheric Watch and ACTRIS programs since 2016 and is located near the summit of Mt. Helmos at the heart of the Peloponnese in Greece. (HAC)^2^, frequently situated in the FT or at the FT/PBL interface^31^, is at a cross-road of different airmasses, each of which is characterized as different INP sources^32^. The observational setup of CALISHTO is presented in the Supplementary Fig. S1, and also elsewhere in detail^32^, including high resolution measurements of in-situ INPs (~6‒7 min), microphysical and chemical aerosol properties at (HAC)^2^, remote sensing measurements conducted at Vathia Lakka (VL), a site located lower ~0.5 km than (HAC)^2^, and back trajectory analysis for calculating the origin of airmasses sampled at (HAC)^2^. Timeseries of measurement results are presented and introduced in Fig. S2 and Text S2 in the Supplementary Material. The PBLH is determined by a wind lidar at VL and expressed as the vertical distance between VL and the FT/PBL interface^31^. The PBLH is used to determine whether (HAC)^2^ is in the PBL, given that (HAC)^2^ is located 500 m above VL (illustrated in Supplementary Fig. S1). Measurements of aerosol properties and airmass characteristics at the (HAC)^2^ can also be used to determine whether the site is in the PBL or the FT when PBLH from lidar remote sensing measurements is not available. For example, the site is in the PBL (for 82.5% of the period) if the concentration of particles between 95 nm and 800 nm (SMPS_>95nm,<800nm_, measured by a TSI Inc., model 3938 scanning mobility particle sizer; SMPS) exceeds a threshold value of 100 std cm^−3^ ^32,34^. A thorough evaluation against other metrics and the PBLH^31,35^ further confirms the universality of the 100 std cm^−3^ threshold, likely because aerosols in the FT are highly diluted after transporting from far-away PBL sources^31^ and generally are much lower in concentration compared to PBL-influenced airmasses.

**Fig. 1:**
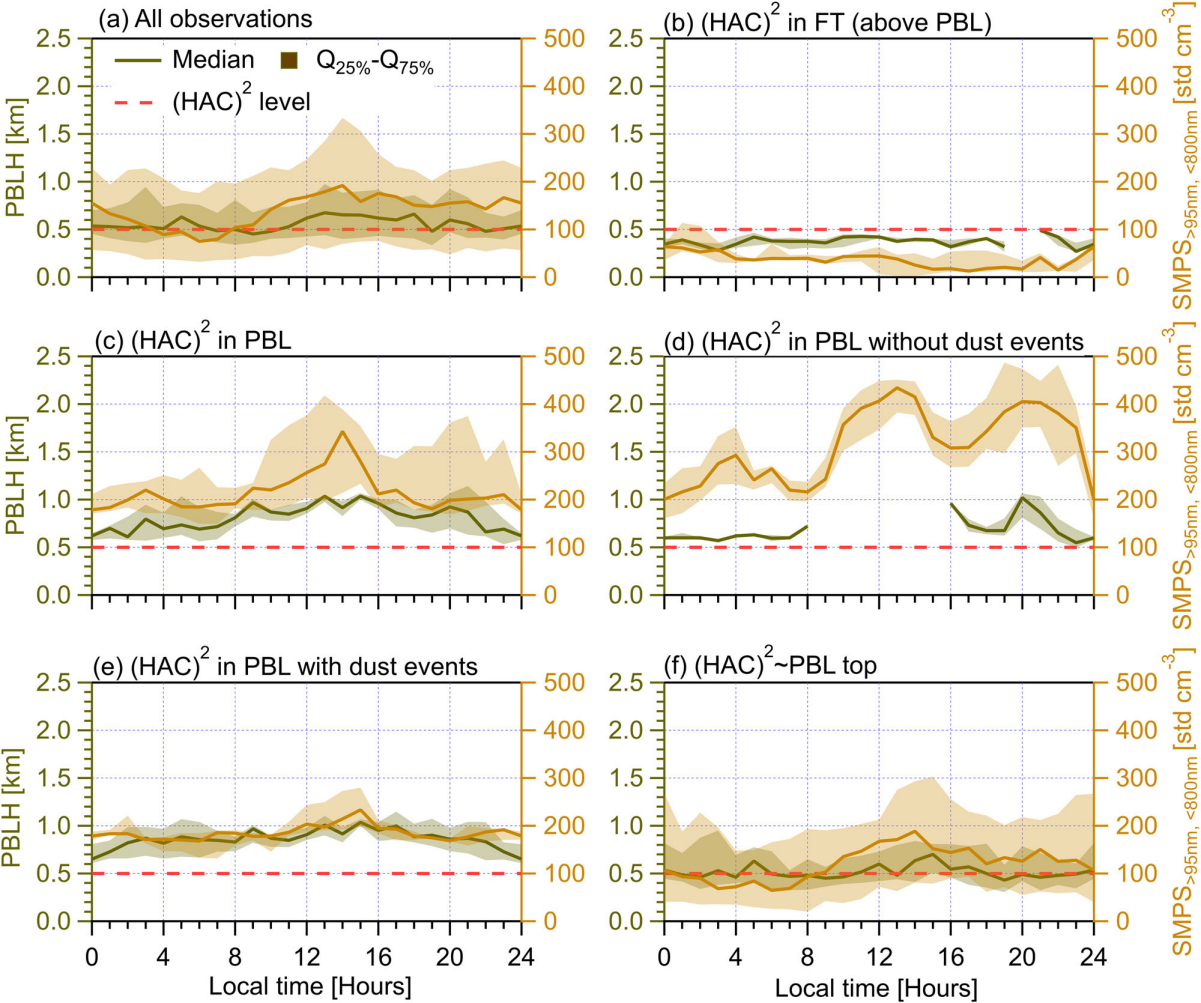
Diurnal cycles of PBLH measured by wind lidar^31^ at the VL site (on the left axis) and corresponding diurnal cycles of the number concentration of particles with diameter between 95 nm and 800 nm (SMPS_>95nm, <800nm_) measured at (HAC)^2^ (on the right axis). Solid lines indicate median values and the shading area around the median line shows the range between 25th and 75th quartiles. The horizontal dashed red line indicates both the altitude difference between (HAC)^2^ and the lidar (~0.5 km) and the threshold particle number value of 100 std cm^−3^ ^31,35^. Different (HAC)^2^ atmospheric conditions are classified in different panels. **a** All observations during the campaign. **b** (HAC)^2^ in the FT throughout the day. **c** (HAC)^2^ in the PBL throughout the day. **d** (HAC)^2^ in the PBL throughout the day without dust event influence. **e** (HAC)^2^ in the PBL throughout the day with dust event influence. **f** (HAC)^2^ for days with both PBL and FT influences. The data points of each (HAC)^2^ position scenario are resampled for every 20 min and each panel shows period cycles of 24 h starting at 00:00 UTC + 2 (local time) of the day.

Depending on the (HAC)^2^ position with respect to the FT/PBL interface (i.e., its relation to the PBLH), we classify a number of atmospheric state scenarios including: (HAC)^2^ in FT throughout the day (Fig. 1b for PBLH < 0.5 km, 5 days), (HAC)^2^ in PBL throughout the day (Fig. 1c for PBLH > 0.5 km, 9 days), and (HAC)^2^ partially in the PBL throughout the day (Fig. 1f, 30 days), which is termed (HAC)^2^ ~ PBL means (HAC)^2^ fluctuates around PBL/FT interface. Continuous dust events were recorded between November 5 and 9 (Supplementary Fig. S2). Thus, we further divide the scenario of (HAC)^2^ in the PBL throughout the day into two cases, i.e., without (Fig. 1d, 4 days) and with (Fig. 1e, 5 days) dust events – to investigate the effect of Saharan dust events on INP variability. The presence of dust is characterized by significant increases in coarse-sized particle (>2.5 μm) concentrations, low Ångstrӧm exponents ( < 1)^32^ from optical scattering measurement and the spatiotemporal dust mass distribution predicted by modeling experiments (Supplementary Fig. S2c, d, f respectively). For the scenario of (HAC)^2^ in FT throughout the day (Fig. 1b), no appreciable diurnal cycle is seen for both PBLH and SMPS_>95nm,<800nm_, while a clear diurnal cycle is observed for days when the (HAC)^2^ is in the PBL (Fig. 1c), showing the maximum in the afternoon. The drivers of PBLH diurnal cycles are discussed in Supplementary Text S3 based on results in Figs. S3 to S6. We note that larger SMPS_>95nm,<800nm_ concentrations of non-dust days (Fig. 1d) compared with those of dust days (Fig. 1e) are because of differences in aerosol sources. On non-dust days, (HAC)^2^ is influenced by continental aerosols enriched in fine mode particles (generally <500 nm, see Figs. S7 and S8), whereas the presence of dust events on dust days tends to shift the size distribution of aerosols to larger sizes with a depletion in fine mode particles according to parallel studies on the aerosol sources^32^ and PBLH^36^ at (HAC)^2^ during CALISHTO campaign.

### INP diurnal cycles under different atmospheric conditions

**Fig. 2:**
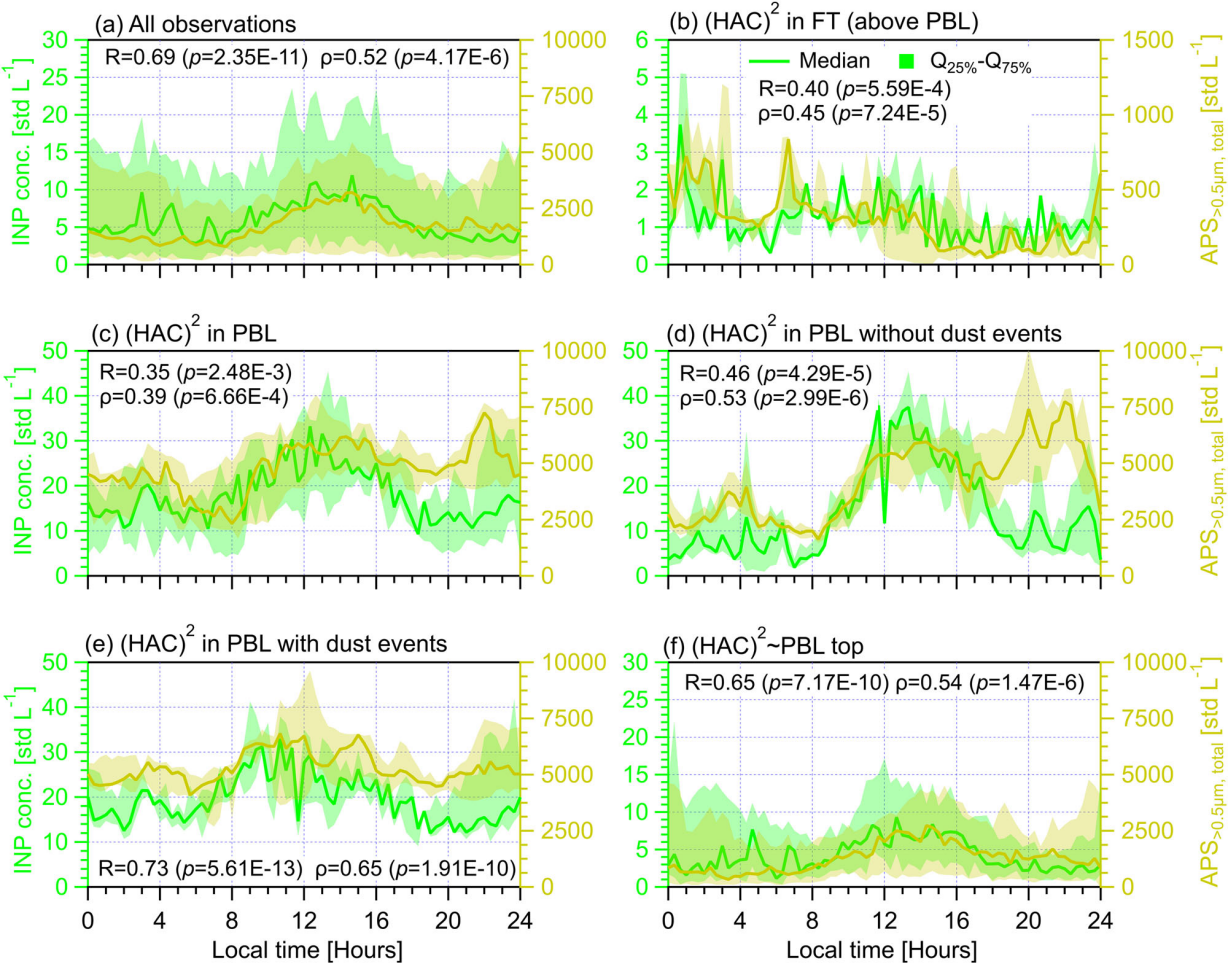
Diurnal cycles of INP (tested at *T* = − 25.2 ± 1.4 °C, on the left axis) and APS_>0.5μm, total_ (the total number concentration of particles between 0.5 and 20 μm measured by an Aerodynamic Particle Sizer, on the right axis) measured at (HAC)^2^ under different atmospheric conditions. Solid lines indicate median values and the shading area around the median line shows the range between 25th and 75th quartiles. Different (HAC)^2^ atmospheric conditions are classified in different panels. **a** All observations during the campaign. **b** For days only in the FT. **c** For days only in the PBL. **d** Days in the PBL without dust events. **e** Days in the PBL with dust events. **f** Days not exclusively in the PBL or FT. The data points of each scenario are resampled for every 20 min and each panel shows a cycle period of 24 h starting at 00:00 UTC + 2 (local time) of the day. The Pearson correlation coefficient (*R*) and Spearman’s rank coefficient (ρ), as well as corresponding *p* values, are provided to evaluate the correlation between INP concentration and APS_>0.5μm, total_. The *P* value is the probability of obtaining an *R* (ρ) value no smaller than the true *R* (ρ) value if there is no liner correlation between INP and APS_>0.5μm, total_. The number of data points (*n*) for each case of above statistical analysis is 72.

The INP number concentration at (HAC)^2^ was determined by a portable ice nucleation experiment (PINE) at *T* = −25.2 ± 1.4 °C in the mixed phase cloud regime (Fig. S2b). PINE samples aerosols from an omnidirectional total inlet and tests INPs in all freezing mode by addressing supersaturated conditions with respect to water^32^. Analysis of their concentration over 24 h periods (e.g., Fig. 2) reveals diurnal periodicities depending on the PBL condition. As shown in Fig. 2a, median INP concentration overall increases from 3.0 std L^−1^ during daybreak to a maximum of 12.0 std L^−1^ in the early afternoon (12:00‒15:00) and subsequently decreases to approximately 3.0 std L^−1^. Then it remains a fairly constant level of 3.0‒4.0 std L^−1^ throughout the evening until the early morning (at 3:00), after which the concentration occasionally spikes up to ~10.0 std L^−1^ (Fig. 2a). Figure 2 shows that the INP diurnal patterns for days influenced by PBL airmasses (Fig. 2c–f) are generally analogous to the overall INP diurnal periodicity presented in Fig. 2a but show different maximum and minimum values. INPs for days only in the FT (Fig. 2b), however, do not exhibit such a diurnal cycle but generally show median concentration values less than 3.0 std L^−1^ throughout the day. The above results suggest that the source of INPs observed at (HAC)^2^ originates primarily from the PBL, and that the diurnal cycle of INPs is driven by the influx of aerosol particles from the PBL to the site. This is consistent with Brunner et al.^23^, who found an absence of INP diurnal cycles in the FT, but a clear diurnal cycle is observed when PBL airmasses are available.

### The dependence of INP diurnal cycles on aerosol particle size

**Fig. 3:**
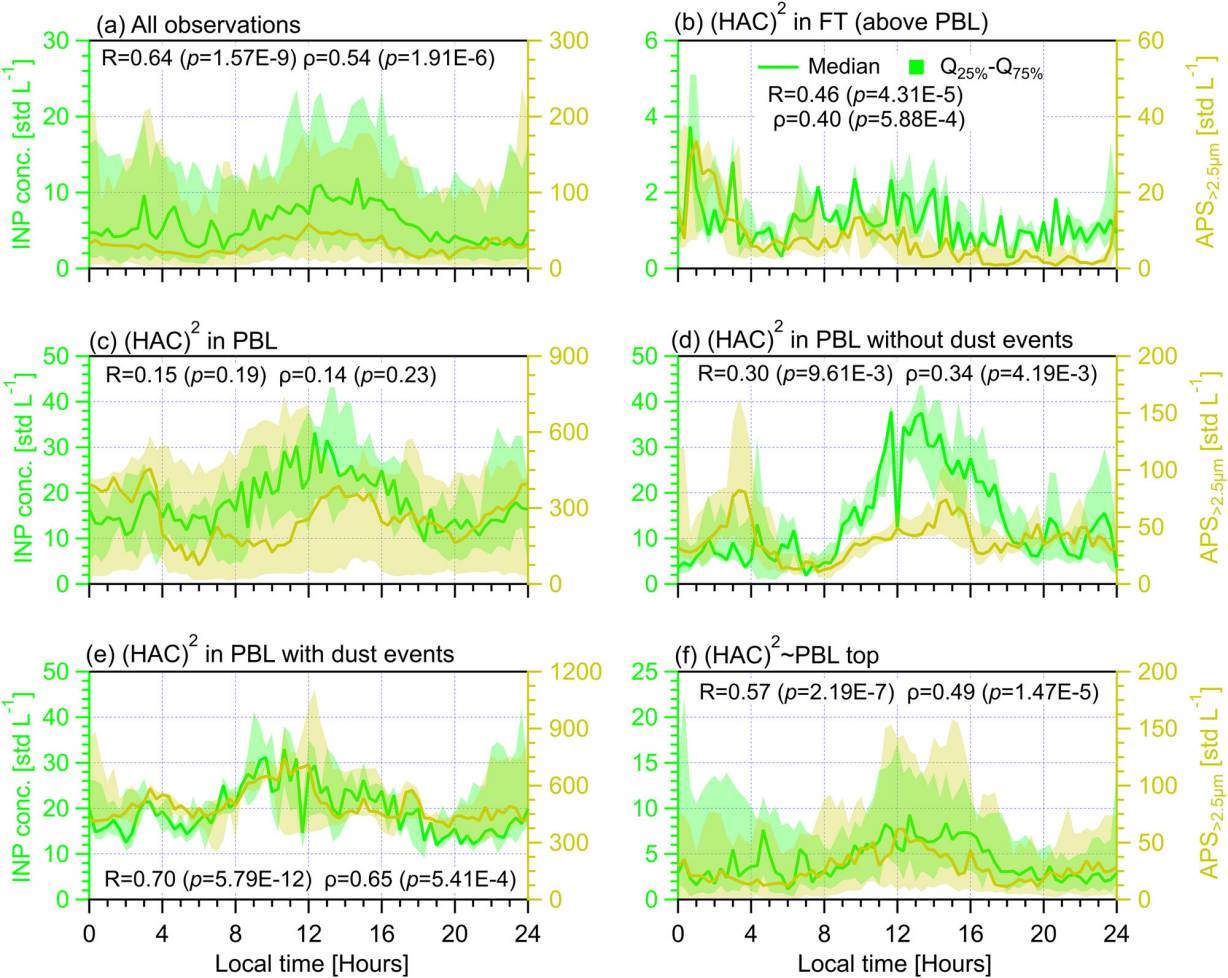
Diurnal cycles of INP (tested at *T* = −25.2 ± 1.4 °C, on the left axis) and the concentration of aerosol particles between 2.5 and 20 μm (APS_>2.5μm_, on the right axis) measured at (HAC)^2^ under different atmospheric conditions. Solid lines indicate median values and the shading area around the median line shows the range between 25th and 75th quartiles. Different (HAC)^2^ atmospheric conditions are classified in different panels. **a** All observations during the campaign. **b** For days only in the FT. **c** For days only in the PBL. **d** Days in the PBL without dust events. **e** Days in the PBL with dust events. **f** Days not exclusively in the PBL or FT. The data points of each scenario are resampled for every 20 min and each panel shows a cycle period of 24 h starting at 00:00 UTC + 2 (local time) of the day. The Pearson correlation coefficient (*R*) and Spearman’s rank coefficient (ρ), as well as corresponding *p* values, are provided to evaluate the correlation between INP concentration and APS_>2.5μm_. The *p* value is the probability of obtaining an *R* (ρ) value no smaller than the true *R* (ρ) value if there is no liner correlation between INPs and APS_>2.5μm_. The number of data points (*n*) for each case of above statistical analysis is 72.

To study the correlations between the diurnal variabilities of INPs and aerosol particle sizes, we compare the INP diurnal cycles with diurnal changes of the number concentration of total aerosol particles in different size ranges measured by a SMPS (10‒800 nm, electrical mobility diameter) and an aerodynamic particle sizer (APS, 0.5‒20 μm, aerodynamic diameter). Particles in different size ranges used to compare with INPs include SMPS+APS_total_ (0.01‒20 μm, Fig. S7), APS_>0.5μm, total_ (0.5‒20 μm, Fig. 2), SMPS_<500nm_ (Fig. S8), APS_>0.5μm,<1.0μm_ (Fig. S9), APS_>1.0μm,<1.5μm_ (Fig. S10), APS_>1.5μm,<2.0μm_ (Fig. S11), APS_>2.0μm,<2.5μm_ (Fig. S12) and APS_>2.5μm_ (Fig. 3). The definition of particles in each size range is provided in the footnote of Table 1. SMPS+APS_total_ is superimposed by both SMPS and APS results^32,37^. Additionally, scatter plots comparing INP number concentrations with meteorological parameters (ambient *T*, i.e., *T*_ambient_, horizontal wind velocity and direction) and different aerosol properties (fluorescent and optical properties, as well as eBC mass concentration) under different PBL conditions are provided in Fig. S13 in Supplementary S5. Also, the correlation between INP diurnal cycles and eBC mass concentration and aerosol optical properties are also examined respectively (Figs. S14 to S16 in Supplementary S6).

**Table 1:** The Pearson correlation coefficient (*R*) and Spearman’s rank coefficient (ρ) for the relationship evaluation between diurnal INP median number concentration and the median number concentration of aerosol particles with different sizes under different atmospheric conditions

Scenarios	All observations		(HAC)^2^ in FT (above PBL)		(HAC)^2^ in PBL		(HAC)^2^ in PBL without dust events		(HAC)^2^ in PBL with dust events		(HAC)^2^ ~ PBL top	
	*R* (*p*)	*ρ* (*p*)	*R* (*p*)	*ρ* (*p*)	*R* (*p*)	*ρ* (*p*)	*R* (*p*)	*ρ* (*p*)	*R* (*p*)	*ρ* (*p*)	*R* (*p*)	*ρ* (*p*)
APS_>0.5μm, total_^a^	**0.69**	**0.52**	**0.40**	**0.45**	**0.35**	**0.39**	**0.46**	**0.53**	**0.73**	**0.65**	**0.65**	**0.54**
	**<0.01**	**0.01**	**<0.01**	**<0.01**	**<0.01**	**<0.01**	**<0.01**	**<0.01**	**<0.01**	**<0.01**	**<0.01**	**<0.01**
SMPS+APS_total_^b^	**0.45**	**0.29**	0.13	0.03	**0.51**	**0.46**	0.05	0.11	0.15	0.23	**0.33**	**0.25**
	**<0.01**	**0.01**	0.27	0.81	**<0.01**	**<0.01**	0.70	0.37	0.20	0.06	**0.05**	**0.04**
SMPS_<500nm_^c^	**0.40**	**0.23**	0.12	0.01	**0.51**	**0.47**	0.04	0.10	0.12	0.18	**0.27**	0.20
	**<0.01**	**0.05**	0.31	0.90	**<0.01**	**<0.01**	0.73	0.40	0.33	0.13	**0.02**	0.09
APS_>0.5μm,<1.0μm_^d^	**0.59**	**0.43**	**0.33**	**0.43**	**0.58**	**0.55**	**0.47**	**0.53**	**0.44**	**0.40**	**0.62**	**0.53**
	**<0.01**	**<0.01**	**0.01**	**<0.01**	**<0.01**	**<0.01**	**<0.01**	**<0.01**	**<0.01**	**<0.01**	**<0.01**	**<0.01**
APS_>1.0μm,<1.5μm_^e^	**0.71**	**0.62**	**0.43**	**0.42**	0.09	0.08	**0.37**	**0.34**	**0.77**	**0.77**	**0.63**	**0.55**
	**<0.01**	**<0.01**	**<0.01**	**<0.01**	0.47	0.48	**<0.01**	**<0.01**	**<0.01**	**<0.01**	**<0.01**	**<0.01**
APS_>1.5μm,<2.0μm_^f^	**0.60**	**0.53**	**0.44**	**0.38**	0.08	0.09	**0.28**	**0.32**	**0.76**	**0.77**	**0.57**	**0.54**
	**<0.01**	**<0.01**	**<0.01**	**<0.01**	0.49	0.46	**0.02**	**0.01**	**<0.01**	**<0.01**	**<0.01**	**<0.01**
APS_>2.0μm,<2.5μm_^g^	**0.58**	**0.53**	**0.43**	**0.35**	0.10	0.09	**0.24**	**0.31**	**0.75**	**0.75**	**0.51**	**0.46**
	**<0.01**	**<0.01**	**<0.01**	**<0.01**	0.41	0.44	**0.05**	**0.01**	**<0.01**	**<0.01**	**<0.01**	**<0.01**
APS_>2.5μm_^h^	**0.64**	**0.54**	**0.46**	**0.40**	0.15	0.14	**0.30**	**0.34**	**0.70**	**0.65**	**0.57**	**0.49**
	**<0.01**	**<0.01**	**<0.01**	**<0.01**	0.19	0.23	**<0.01**	**<0.01**	**<0.01**	**<0.01**	**<0.01**	**<0.01**

A critical *p* value of 0.05 from F-test for *R* and ρ is used to assess the significance level of the relationship. A *p* value smaller than 0.05 suggests that the probability of obtaining an *R* (ρ) value no smaller than the true *R* (ρ) value is less than 5% if there is actually no liner correlation between INPs and the given parameter, thus the calculated *R* (ρ) is of statistical significance. Evaluated significant relationships are indicated in bold. The correlation coefficients are also provided in Figs. 2 and 3 and Figs. S7 to S12 in Supplement S4.

^a^Total particle (0.5–20 μm) number concentration measured APS,

^b^Total particle (0.01–20 μm) number concentration measured by both SMPS and APS,

^c^The number concentration of particles smaller than 500 nm measured by SMPS,

^d^The number concentration of particles between 0.5 and 1.0 μm measured by APS,

^e^The number concentration of particles between 1.0 and 1.5 μm measured by APS,

^f^The number concentration of particles between 1.5 and 2.0 μm measured by APS,

^g^The number concentration of particles between 2.0 and 2.5 μm measured by APS,

^h^The number concentration of particles larger than 2.5 μm measured by APS.

Given the size dependence of INPs^6,38^, APS_>0.5μm, total_ particles are assumed to be major INP contributors in the literature whereas smaller-sized particles (<0.5 μm), which are even not included in some INP parameterizations^7,39,40^, are assumed to be insignificant INPs. Coarse mode particles (e.g., APS_>2.5μm_) are more relevant for dust and bioaerosols and considered with a higher probability of serving as INPs^6,7^. Here, we evaluate the contribution of APS_>0.5μm, total_ and APS_>2.5μm_ particles to the observed INPs under different atmospheric conditions (Figs. 2 and 3) and also discuss the importance of particles in other size ranges (Table 1). The APS_>0.5μm, total_ (Fig. 2a) and APS_>2.5μm_ (Fig. 3a) concentration cycles from all observations show an overall diurnal cycle similar to that of INPs, respectively. Overall concentration diurnal cycles and significant correlations with INPs are also observed for particles in other size ranges presented in Table 1 and Figs. S7a to S12a.

Analogous to the INP data for days only in the FT, none of size-resolved particles presents a diurnal cycle (e.g., Figs. 2b and 3b), but all shows the lowest median value at the same hours compared to the other scenarios influenced by PBL air masses. Notably, Fig. 2b shows an overall constant APS_>0.5μm, total_ to INP concentration ratio (for median values) of approximately 250, while Fig. 3b presents that the difference between APS_>2.5μm_ and INP medians is generally within a factor of 10. Table 1 shows that particles having a size range larger than 0.5 μm have significant (*p* < 0.05) and positive correlations with INPs throughout the day in the FT whereas particles smaller than 0.5 μm (SMPS_<500nm_) show an insignificant role, which highlights the importance of particles larger than 0.5 μm and is consistent with the literature^7,39,40^. The case of SMPS+APS_total_ in the FT (Table 1 and Fig. S7b) presents similar results to that of SMPS_<500nm_, given that SMPS_<500nm_ takes a major fraction of SMPS+APS_total_^32^.

For days when the (HAC)^2^ is only in the PBL, Table 1 shows that both APS_>0.5μm, total_ and SMPS+APS_total_ present significant contributions (*R* = 0.35 and 0.51 respectively) to the observed INP diurnal cycles (also Fig. 2c and Fig. S7c). It also shows that only particles within a size range smaller than 1.0 μm significantly contribute to the INP diurnal cycle, whereas particles with a larger size range (>1.0 μm) are weakly linked to INP variabilities. This may be because fine mode particles have much higher number concentrations than coarse mode particles^32^ and also because particles from various sources that span different size ranges are responsible for the observed INPs in the PBL^32^. Thus, a higher particle number concentration including smaller-sized particles is more associated with INP variability than low number concentrations of larger-sized particles, when aerosol sources affecting the particle population are many. Only when INP sources are further determined, can the importance of larger-sized particles for INP variability be determined, which is demonstrated by the results in Table 1 for the case of (HAC)^2^ in PBL with and without dust events. It shows that only particles in size ranges larger than 0.5 μm have significant correlations with INP diurnal medians while smaller-sized particles (SMPS_<500nm_ and SMPS+APS_total_) are insignificant. This is consistent with the case of (HAC)^2^ in FT (Table 1) and the literature^7,39,40^.

In addition, there are different size dependences for INP variability observed between cases of (HAC)^2^ in PBL with and without dust events. Table 1 shows that with an increasing size range for particles larger than 0.5 μm (from a range of 0.5‒1.0 μm to a range of 2.5‒20 μm), the significance of larger-sized particles become less and less pronounced for non-dust days. Again, this highlights a more important role of high number concentrations particles in regulating INPs in the PBL with continental aerosols^32^, given that continental aerosols generally contain fine particles without dust events. Differently, the case of (HAC)^2^ in PBL with dust events (Table 1) shows the least significant contributions of particles between 0.5 and 1.0 μm, compared to particles in larger size ranges (>1.0 μm), presenting the importance of larger-sized dust particles in regulating INP variabilities. Notably, particles in size ranges larger than 1.0 μm show similar correlation (*R≥*0.70) and significance levels (<0.01) with INP variabilities (Table 1). This indicates dust particles enrich the observed INPs from a size limit of 1.0 μm, which is in agreement with Gao et al.^32^ (a parallel study) who reported a size distribution inflection point at 1.0 μm for aerosols observed at (HAC)^2^ in the PBL during dust events.

For the case of (HAC)^2^ ~ PBL, it shows similar results to the case of all observations for the correlations between INPs and particles in different size ranges (Table 1). Particles from different size ranges play a significant role in regulating the INP diurnal cycle. It also presents a more important role for particles in size ranges larger than 0.5 μm (*R*≥0.51) compared with smaller-sized particles (*R* = 0.27). In brief, we conclude that particles larger than 0.5 μm are generally important contributors to the INP diurnal periodicity for the (HAC)^2^ in the FT or PBL in the absence or presence of dust events. When a mixture of varying INP sources is relevant, the contribution from smaller-sized particles (<0.5 μm) is non-negligible. Also, the different size dependence of INPs observed for (HAC)^2^ in PBL with and without dust events implies different abundance of INP types in each aerosol source.

### The influence of biomass burning particles on INP diurnal variability

APS_>0.5μm, total_ observed on days when the (HAC)^2^ is only in the PBL without dust influence (Fig. 2d) shows an increase in the evening (~20:00‒22:00), however, such an aerosol particle increase does not lead to increased INPs. Note that such a particle concentration spark is only present for particles in a range smaller than 1.5 μm (Figs. S8d to S10d). The presence of those increased particles coincides with the peak values of elemental black carbon (eBC) mass concentration (measured by an aethalometer, AE31, Magee Scientific, US) in the evening (Fig. S14d in Supplementary S6), probably due to increased use of fossil fuels for heating during the cold days at the nearby village of Kalavryta (Supplementary Fig. S4d for low *T*_ambient_). This is also supported by a positive and significant correlation between eBC mass concentration and the number concentration of total (observed by both SMPS and APS) particles smaller than 1.5 μm (*R* = 0.44 (*p* = 8.31E − 6) and *ρ* = 0.68 (*p* = 4.63E − 14)) for the (HAC)^2^ in the PBL without dust events but a negative and insignificant correlation with larger-sized (>1.5 μm) particles (*R* = − 0.11 (*p* = 0.28) and *ρ* = −0.09 (*p* = 0.39)). Particles containing eBC masses or black carbon particles are poor INPs in the MPC regime^41^. Thus, those eBC containing particles (<1.5 μm) do not lead to INP increases. This is also similar to the results reported by Brunner et al.^23^ for Jungfraujoch. Therefore, contributions from eBC-containing particles are negligible to INPs tested at *T* = − 25.2 ± 1.4 °C in this study.

### The dependence of INP diurnal cycles on fluorescent biological aerosol particles in different size ranges

**Fig. 4:**
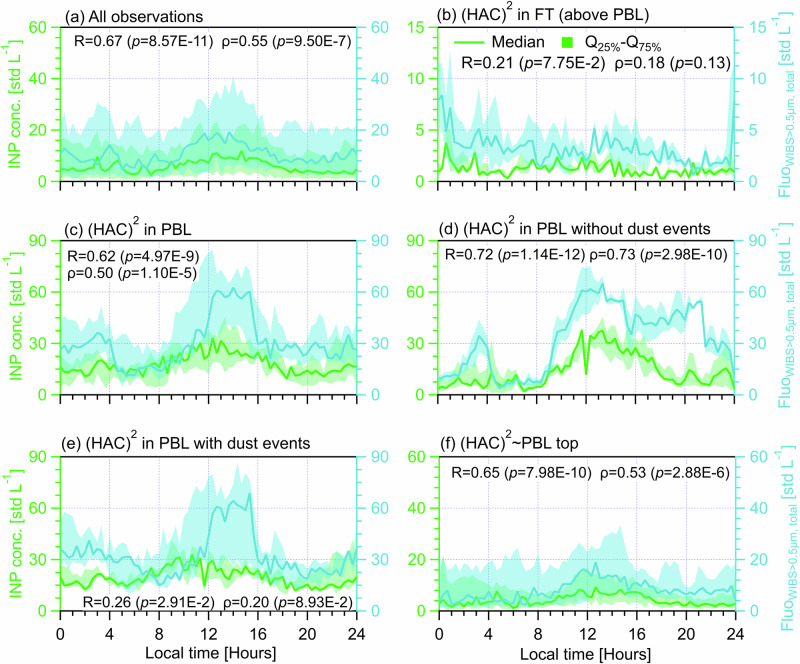
Diurnal cycles of INP (tested at *T* = −25.2 ± 1.4 °C, on the left axis) and Fluo_WIBS>0.5μm, total_ (the number concentration of particles between 0.5 and 30 μm showing fluorescence in any of three channels of a wideband integrated bioaerosol sensor (WIBS), on the right axis) measured at (HAC)^2^ under different atmospheric conditions. Solid lines indicate median values and the shading area around the median line shows the range between 25th and 75th quartiles. Different (HAC)^2^ atmospheric conditions are classified in different panels. **a** All observations during the campaign. **b** For days only in the FT. **c** For days only in the PBL. **d** Days in the PBL without dust events. **e** Days in the PBL with dust events. **f** Days not exclusively in the PBL or FT. The data points of scenario are resampled for every 20 min and each panel shows a cycle period of 24 h starting at 00:00 UTC + 2 (local time) of the day. The Pearson correlation coefficient (*R*) and Spearman’s rank coefficient (ρ), as well as corresponding *p* values, are provided to evaluate the correlation between INP concentration and Fluo_WIBS>0.5μm, total_. The *p* value is the probability of obtaining an *R* (ρ) value no smaller than the true *R* (ρ) value if there is no liner correlation between INPs and Fluo_WIBS>0.5μm, total_. The number of data points (*n*) for each case of above statistical analysis is 72.

Particles of biological origin (even if not all INP-active) are key contributors to INPs in the MPC regime, particularly for *T* > −15 °C^42,43^. Also, dust particles, as important contributors to INPs for *T* < −15 °C^42,43^, are often found to be associated with biological material^44^, like airborne bacteria coexisting with Saharan dust particles^45^ and soil dust rich in biological materials at source^46^. Fluorescence is an important property indicating particles carrying biological materials, although some particles of non-biological origin can also fluoresce^21,47^. Fluorescent particles have been frequently viewed as a lower-limit proxy for biological particles^48^, called fluorescent biological aerosol particles (FBAPs)^47^. Here, a wideband integrated bioaerosol sensor (WIBS, WIBS-5/NEO, Droplet Measurement Technologies, LLC. US) was used to measure the number concentration of FBAPs at (HAC)^2^ and record the optical size of the particle (0.5‒30 μm)^32,47^. Particles fluorescing in any one of the three WIBS fluorescent channels is classified as Fluo_WIBS_ particles^32^ which includes all FBAPs detectable by a WIBS. FBAPs are demonstrated to be significant INP predictors during the CALISHTO^32^ and other field campaigns in other regions^24,39^. Hence, we compare the diurnal cycles of both INPs and total FBAPs, represented by Fluo_WIBS>0.5μm, total_, to investigate the role of bioaerosols in INP variabilities (Fig. 4). Also, we investigate the contribution of FABPs in different size ranges to the observed INP diurnal cycles, including Fluo_WIBS>0.5μm,<1.0 μm_ (Fig. S17), Fluo_WIBS>1.0μm,<1.5μm_ (Fig. S18), Fluo_WIBS>1.5μm,<2.0μm_ (Fig. S19), Fluo_WIBS>2.0μm,<2.5μm_ (Fig. S20) and Fluo_WIBS>2.5μm_ (Fig. S21). The calculated correlation coefficients between diurnal medians INPs and Fluo_WIBS_ particles in different size ranges are summarized in Table 2.

**Table 2:** The Pearson correlation coefficient (*R*) and Spearman’s rank coefficient (ρ) for the relationship evaluation between diurnal INP median number concentration and the median number concentration of fluorescent biological aerosol particles (FBAPs) with different sizes under different atmospheric conditions

Scenarios	All observations		(HAC)^2^ in FT (above PBL)		(HAC)^2^ in PBL		(HAC)^2^ in PBL without dust events		(HAC)^2^ in PBL with dust events		(HAC)^2^ ~ PBL top	
	*R* (*p*)	*ρ* (*p*)	*R* (*p*)	*ρ* (*p*)	*R* (*p*)	*ρ* (*p*)	*R* (*p*)	*ρ* (*p*)	*R* (*p*)	*ρ* (*p*)	*R* (*p*)	*ρ* (*p*)
Fluo_WIBS>0.5μm, total_^a^	**0.67**	**0.55**	0.21	0.18	**0.62**	**0.50**	**0.72**	**0.73**	**0.26**	0.20	**0.65**	**0.53**
	**<0.01**	**<0.01**	0.08	0.13	**<0.01**	**<0.01**	**<0.01**	**<0.01**	**0.03**	0.09	**<0.01**	**<0.01**
Fluo_WIBS>0.5μm, <1.0μm_^b^	**0.43**	**0.39**	−0.06	−0.05	**0.59**	**0.46**	**0.70**	**0.71**	0.17	0.21	**0.34**	**0.26**
	**<0.01**	**<0.01**	0.62	0.68	**<0.01**	**<0.01**	**<0.01**	**<0.01**	0.17	0.07	**<0.01**	**0.03**
Fluo_WIBS>1.0μm, <1.5μm_^c^	**0.42**	**0.42**	−0.06	−0.05	**0.62**	**0.56**	**0.58**	**0.62**	**0.43**	**0.53**	**0.38**	**0.41**
	**<0.01**	**<0.01**	0.62	0.68	**<0.01**	**<0.01**	**<0.01**	**<0.01**	**<0.01**	**<0.01**	**<0.01**	**<0.01**
Fluo_WIBS>1.5μm, <2.0μm_^d^	**0.64**	**0.60**	0.07	0.01	**0.68**	**0.61**	**0.67**	**0.67**	**0.39**	**0.49**	**0.42**	**0.46**
	**<0.01**	**<0.01**	0.56	0.95	**<0.01**	**<0.01**	**<0.01**	**<0.01**	**<0.01**	**<0.01**	**<0.01**	**<0.01**
Fluo_WIBS>2.0μm, <2.5μm_^e^	**0.41**	**0.43**	0.02	−0.03	**0.70**	**0.59**	**0.75**	**0.69**	**0.39**	**0.41**	**0.29**	**0.30**
	**<0.01**	**<0.01**	0.88	0.78	**<0.01**	**<0.01**	**<0.01**	**<0.01**	**<0.01**	**<0.01**	**0.01**	**0.01**
Fluo_WIBS>2.5μm_^f^	**0.65**	**0.58**	0.20	0.18	**0.42**	**0.38**	**0.74**	**0.70**	0.21	0.17	**0.61**	**0.56**
	**<0.01**	**<0.01**	0.09	0.13	**<0.01**	**<0.01**	**<0.01**	**<0.01**	0.07	0.16	**<0.01**	**<0.01**

A critical *p* value of 0.05 from F-test for *R* (ρ) is used to assess the significance level of the relationship. A *p* value smaller than 0.05 suggests that the probability of obtaining an *R* (ρ) value no smaller than the true *R* (ρ) value is less than 5% if there is actually no liner correlation between INPs and the given parameter, thus the calculated *R* (ρ) is of statistical significance. Evaluated significant relationships are indicated in bold. The correlation coefficients are also provided in Fig. 4 in the main text and Figs. S17 to S21 in Supplement S7.

^a^Total particle (0.5–30 μm) number concentration measured by WIBS,

^b^The number concentration of particles between 0.5 and 1.0 μm measured by WIBS,

^c^The number concentration of particles between 1.0 and 1.5 μm measured by WIBS,

^d^The number concentration of particles between 1.5 and 2.0 μm measured by WIBS,

^e^The number concentration of particles between 2.0 and 2.5 μm measured by WIBS,

^f^The number concentration of particles larger than 2.5 μm measured by WIBS.

Table 2 shows an overall medium and significant correlations between the diurnal cycles of INPs and FABPs in all calculated size ranges (the column of all observations), suggesting FBAPs in all size ranges contribute to the observed INPs. Of all size ranges, Fluo_WIBS>1.5μm, <2.0μm_ and Fluo_WIBS>2.5μm_ are more important INP contributors, showing stronger correlations with INPs (*R*≥0.64) than other size ranges. Fluo_WIBS>1.5μm,<2.0μm_ may coincide with large-sized bacteria or small-sized fungal spores (<2.0 μm), and Fluo_WIBS>2.5μm_ is likely related to fungal spores or larger-sized pollen fragments^49,50^, all of which are often found as effective INPs^51–53^. For the case of (HAC)^2^ in FT, FBAPs in all size ranges show weak (both *R* and *ρ* < 0.2) and insignificant correlations with the observed INPs (Table 2). Nevertheless, the INP diurnal medians in the FT are approximate to those of Fluo_WIBS>0.5μm, total_ (within a factor of 3, see Fig. 4b), particularly similar to those of Fluo_WIBS>2.5μm_ (in Fig. S21b). This suggests scarce FABPs in the FT are non-negligible INP sources, supported by a strong correlation between the scatter plots of all data points of INPs and Fluo_WIBS>0.5μm, total_ (*ρ* = 0.63, *p* = 6.45E-10, see Fig. S13h).

When the (HAC)^2^ is located in the PBL, FBAPs in all calculated size ranges show medium or stronger (e.g., Fluo_WIBS>2.0μm,<2.5μm_) correlations with INP diurnal median values (Table 2). Compared with the combined case for (HAC)^2^ in PBL regardless of the effect of dust events, FABPs on non-dust days in each size range generally show much stronger (R and ρ ≥ 0.6) correlations with INP diurnal medians, while they show less pronounced correlations (*R* and *ρ* < 0.5) during dust days. It suggests a non-negligible role of FBAPs as INP contributors during Saharan dust events. Notably, this indicates a more important role of FABPs in contributing INPs when continental and local aerosols are more dominant INP sources at the (HAC)^2^. Altogether, this suggests that FBAPs relevant for INPs generally originate from PBL aerosols in the absence of dust; during dust events, only FABPs between 1.0 and 2.5 μm are moderately and significantly correlated with the observed INPs, likely because smaller-sized FBAPs have a longer residence time and are easier to be mixed with transported dust aerosols. However, much smaller-sized Fluo_WIBS>0.5μm, <1.0 μm_ particles show a weak and insignificant correlation with INPs during dust days (Table 2 and Fig. S17e) but a strong correlation during non-dust days (Fig. S 17d). Likely, those small-sized particles relevant for INPs may be related to ice-nucleating bacteria (later discussed with results presented in Table 3 and Supplementary S8) – as local and continental aerosols may contain more bacteria whereas transported Saharan dust across less polluted Mediterranean may carry relatively less. Additionally, we note that eBC-containing particles as interfering FBAPs^47^ may be responsible for the small peak of Fluo_WIBS>0.5μm, total_ at 20:00-22:00 h in Fig. 4d, which is only pronounced for FBAPs smaller than 1.5 μm (Figs. S17d and 18d). Again, the results in the case of (HAC)^2^ ~ PBL (i.e., (HAC)^2^ fluctuates around PB/FT interface) are similar to the case of all observations, possibly because both cases contain a mixture of varying types of FABPs. In summary, Table 2 shows that both FBAPs and dust particles are important sources of INPs, but INP variabilities on non-dust days are more dependent on FBAPs whereas FBAPs are less important (but still significant, e.g., Fluo_WIBS_ between 1.0 and 2.5 μm) INP contributors during dust events overwhelmed by dust particles.

**Table 3:** The Pearson correlation coefficient (*R*) and Spearman’s rank coefficient (ρ) for the relationship evaluation between diurnal INP median number concentration and the median number concentration of different types of fluorescent biological aerosol particles (FBAPs) under different atmospheric conditions

Scenarios	All observations		(HAC)^2^ in FT (above PBL)		(HAC)^2^ in PBL		(HAC)^2^ in PBL without dust events		(HAC)^2^ in PBL with dust events		(HAC)^2^ ~ PBL top	
	*R* (*p*)	*ρ* (*p*)	*R* (*p*)	*ρ* (*p*)	*R* (*p*)	*ρ* (*p*)	*R* (*p*)	*ρ* (*p*)	*R* (*p*)	*ρ* (*p*)	*R* (*p*)	*ρ* (*p*)
A_WIBS_^a^	**0.65**	**0.67**	0.22	0.24	**0.68**	**0.61**	**0.81**	**0.77**	**0.40**	**0.43**	**0.44**	**0.50**
	**<0.01**	**<0.01**	0.08	0.05	**<0.01**	**<0.01**	**<0.01**	**<0.01**	**<0.01**	**<0.01**	**<0.01**	**<0.01**
B_WIBS_^b^	**0.51**	**0.39**	−0.10	−0.05	**0.64**	**0.47**	**0.63**	**0.66**	**0.29**	**0.33**	**0.36**	**0.25**
	**<0.01**	**<0.01**	0.40	0.67	**<0.01**	**<0.01**	**<0.01**	**<0.01**	**0.01**	**0.01**	**<0.01**	**0.04**
C_WIBS_^c^	**0.53**	**0.54**	0.15	0.08	**0.53**	**0.49**	**0.70**	**0.69**	**0.32**	**0.28**	**0.28**	**0.28**
	**<0.01**	**<0.01**	0.20	0.52	**<0.01**	**<0.01**	**<0.01**	**<0.01**	**<0.01**	**0.02**	**0.02**	**0.02**
AB_WIBS_^d^	**0.45**	**0.70**	−0.09	−0.18	**0.59**	**0.44**	**0.65**	**0.68**	**0.32**	**0.36**	**0.41**	**0.65**
	**<0.01**	**<0.01**	0.53	0.19	**<0.01**	**<0.01**	**<0.01**	**<0.01**	**0.01**	**<0.01**	**<0.01**	**<0.01**
AC_WIBS_^e^	**0.38**	**0.51**	−0.07	0.12	**0.33**	**0.47**	**0.51**	**0.71**	**0.28**	0.22	0.12	0.22
	**<0.01**	**<0.01**	0.72	0.57	**0.01**	**<0.01**	**<0.01**	**<0.01**	**0.02**	0.06	0.32	0.06
BC_WIBS_^f^	**0.58**	**0.51**	0.10	0.05	**0.55**	**0.42**	**0.70**	**0.72**	0.16	0.15	**0.49**	**0.44**
	**<0.01**	**<0.01**	0.40	0.71	**<0.01**	**<0.01**	**<0.01**	**<0.01**	0.18	0.22	**<0.01**	**<0.01**
ABC_WIBS_^g^	**0.52**	**0.51**	0.08	0.09	**0.67**	**0.55**	**0.78**	**0.72**	**0.40**	**0.40**	**0.44**	**0.52**
	**<0.01**	**<0.01**	0.48	0.47	**<0.01**	**<0.01**	**<0.01**	**<0.01**	**<0.01**	**<0.01**	**<0.01**	**<0.01**

**Fig. 5:**
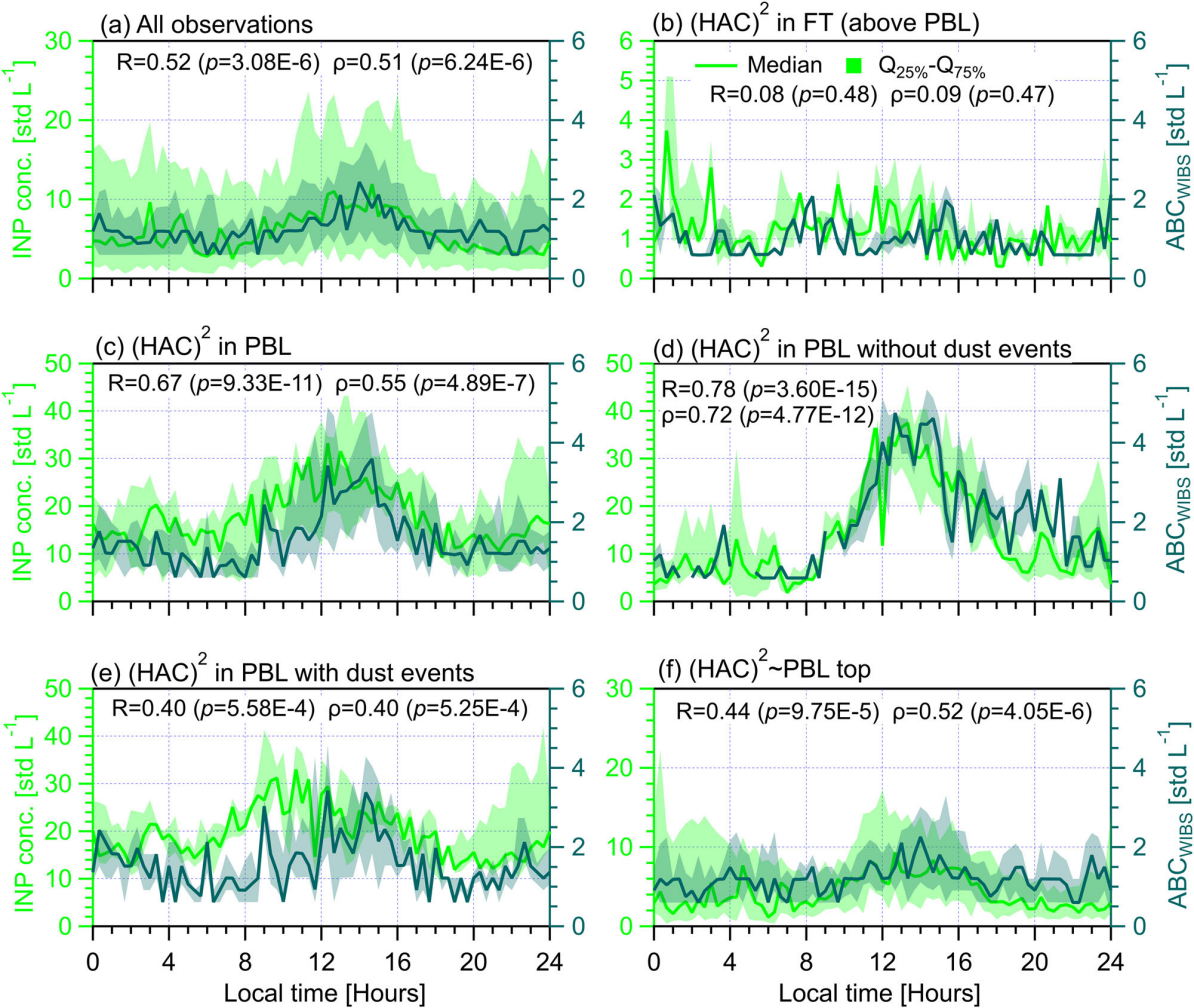
Diurnal cycles of INP (tested at *T* = −25.2 ± 1.4 °C, on the left axis) and ABC_WIBS_ (the number concentration of particles between 0.5 and 30 μm showing fluorescence in all three WIBS channels, on the right axis) measured at (HAC)^2^ under different atmospheric conditions. Solid lines indicate median values and the shading area around the median line shows the range between 25th and 75th quartiles. Different (HAC)^2^ atmospheric conditions are classified in different panels. **a** All observations during the campaign. **b** For days only in the FT. **c** For days only in the PBL. **d** Days in the PBL without dust events. **e** Days in the PBL with dust events. **f** Days not exclusively in the PBL or FT. The data points of scenario are resampled for every 20 min and each panel shows a cycle period of 24 h starting at 00:00 UTC + 2 (local time) of the day. The Pearson correlation coefficient (*R*) and Spearman’s rank coefficient (ρ), as well as corresponding *p* values, are provided to evaluate the correlation between INP concentration and ABC_WIBS_. The *p* value is the probability of obtaining an *R* (ρ) value no smaller than the true *R* (ρ) value if there is no liner correlation between INPs and ABC_WIBS_. The number of data points (*n*) for each case of above statistical analysis is 72.

A critical *p* value of 0.05 from F-test for *R* (ρ) is used to assess the significance level of the relationship. A *p* value smaller than 0.05 suggests that the probability of obtaining an *R* (ρ) value no smaller than the true *R* (ρ) value is less than 5% if there is actually no liner correlation between INPs and the given parameter, thus the calculated *R* (ρ) is of statistical significance. Evaluated significant relationships are indicated in bold. The correlation coefficients are also provided in Fig. 5 in the main text and Figs. S22 to S27 in Supplement S8.

^a^The total number concentration of particles showing fluoresce only in WIBS FL1 channel,

^b^The total number concentration of particles showing fluoresce only in WIBS FL2 channel,

^c^The total number concentration of particles showing fluoresce only in WIBS FL3 channel,

^d^The total number concentration of particles showing fluoresce only in both WIBS FL1 and FL2 channels,

^e^The total number concentration of particles showing fluoresce only in both WIBS FL1 and FL3 channels,

^f^The total number concentration of particles showing fluoresce only in both WIBS FL2 and FL3 channels,

^g^The total number concentration of particles showing fluoresce only in all WIBS FL1, FL2 and FL3 channels.

### The dependence of INP diurnal cycles on different types of fluorescent biological aerosol particles

Different types of biological particles may exhibit distinct fluorescence characteristics in the WIBS^47^. When considered in our analysis (together with size ranges expected for each population), this can help further understand the specific contribution of each biological type to the observed INPs. A detailed FABP type classification is provided in Table 3. Bacteria show fluorescence dominantly in WIBS FL1 channel and can be recorded as A_WIBS_ (dominant), AB_WIBS_, and ABC_WIBS_ particles around 1.0 μm but generally less than 2.0 μm^49,50^. Fungal spores and fungi are also frequently detected as A_WIBS_ (dominant), AB_WIBS_, and ABC_WIBS_ particles larger than 2.0 μm^47,50^. Intact and fragmented pollen grains are often recognized as C_WIBS_, BC_WIBS_, and ABC_WIBS_ (dominant) particles larger than 2.0 μm^47,50^. ABC_WIBS_ particles are reported as FBAPs measured by a WIBS, with the highest probability of being biological particles^47,50^ and minimal interference from non-biological particles like black carbon and dust^21,47^.

Table 3 shows that all types of FBAPs are moderately or even strongly correlated with overall INP diurnal cycles, likely suggesting that different types of biological particles are relevant contributors to the observed INPs throughout the campaign. Similar results, but with weaker correlations, were observed for the case of (HAC)^2^ fluctuating around the PBL-FT interface. For days when (HAC)^2^ is exclusively in the FT, the diurnal cycle of INPs is not correlated with any types of FBAPs. However, the median number concentration values of INPs are generally within a factor of 3 compared to those of A_WIBS_, B_WIBS_, C_WIBS_, BC_WIBS_ and ABC_WIBS_ particles (Fig. 5b and Supplement S8), suggesting that biological particles in FT airmasses are non-negligible INP sources. For days when (HAC)^2^ resides exclusively in the PBL (Fig. 5 and Supplement S8), the diurnal cycle of all different types of FBAPs is significantly correlated with the corresponding INP cycle (e.g., ABC_WIBS_ *R* = 0.67 and *p* < 0.01 in Fig. 5c), which is stronger on non-dust days (e.g., ABC_WIBS_ with *R* = 0.78 in Fig. 5d) but weaker on dust days (e.g., ABC_WIBS_ with *R* = 0.40 in Fig. 5e). The generally much stronger correlations between the diurnal cycles of INPs and all different types of FBAPs for non-dust days in the PBL compared with those of dust days suggest the overall enrichment of different types of biological particles for non-dust days. Also, it indicates that continental and local aerosols are the primary sources of biological particles but not the transported dust plume, which is consistent with the aerosol source apportionment conducted in a parallel study^32^ and results in Table 2. The above results demonstrate FBAPs are primary drivers of the INP diurnal cycles observed at the (HAC)^2^ in the PBL.

### The influence of dust events on aerosol property and INP diurnal variability

**Fig. 6:**
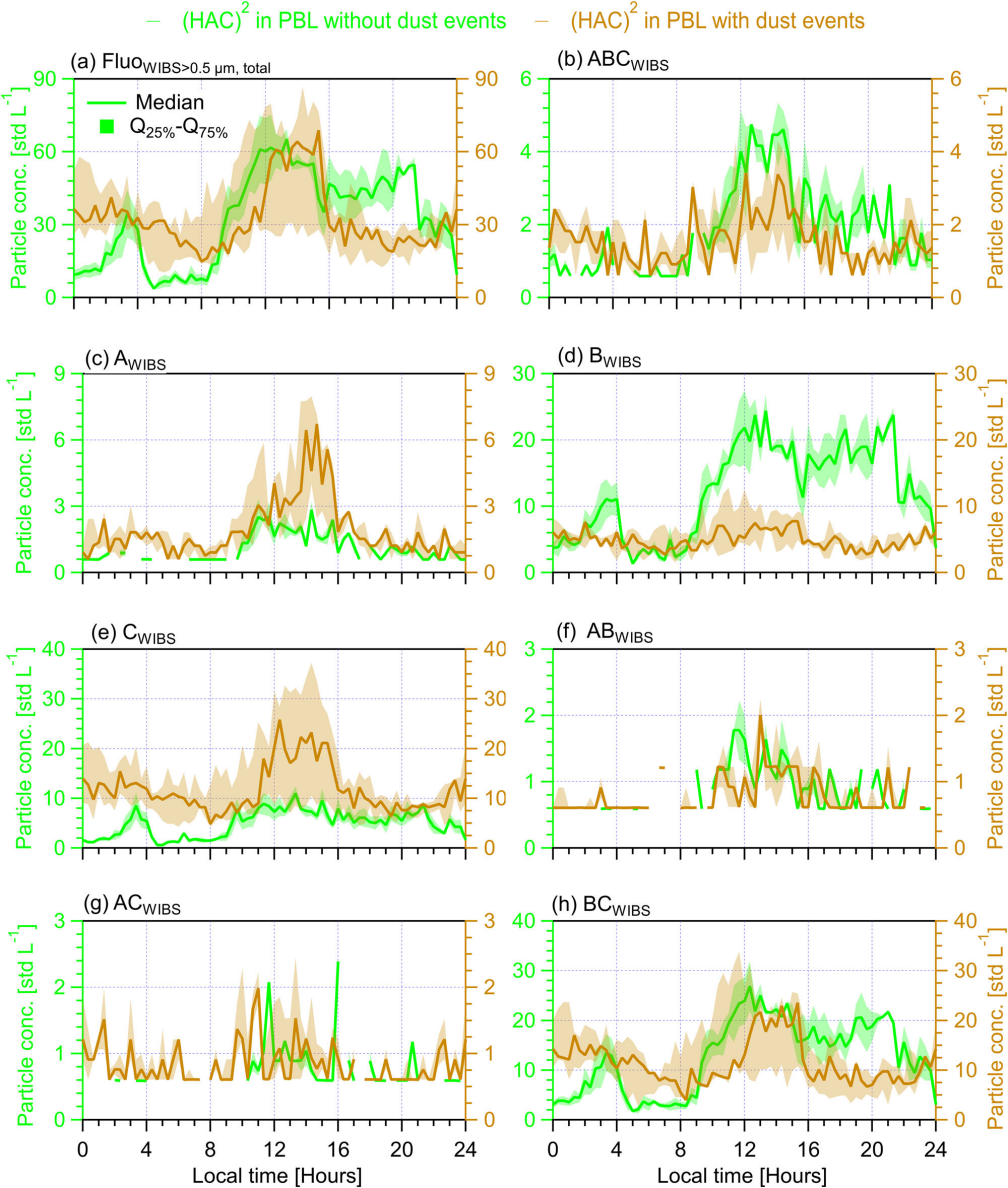
Diurnal cycles of different types of fluorescent biological aerosol particles between 0.5 and 30 µm on days without (left axis) and with (right axis) dust events. Solid lines indicate median values and the shading area around the median line shows the range between 25th and 75th quartiles. **a** Fluo_WIBS>0.5µm,_ _total_, (**b**) ABC_WIBS_, (**c**) A_WIBS_, (**d**) B_WIBS_, (**e**) C_WIBS_, (**f**) AB_WIBS_, (**g**) AC_WIBS_, and (**h**) BC_WIBS_.

Remotely-transported dust plumes modulate aerosols and INPs at the (HAC)^2^ in the PBL. We present the influences of dust events on aerosol properties observed at the (HAC)^2^ by comparing the diurnal cycles of number concentrations of size-resolved total aerosol particles (Fig. S28) and FBAPs (Fig. S29), as well as different types of FBAP (Fig. 6), on non-dust days with dust days. Further discussions on the correlations between aerosol property changes and INP variabilities presented in Figs. 2 to 5 for both cases are conducted. The results in Fig. S28 show that total aerosol particles with size smaller than 0.5 μm during non-dust days are more than those on dust days by at least a factor of 2 (panel c). Dust events enrich particles in size ranges larger than 1.0 μm (panel e to h) for dust days by a factor of 5 ~ 10 compared with those of non-dust days. Similarly, comparing with dust days, Fig. S29 shows that non-dust days contain more FBAPs of size below 2.0 μm (panel b, c and d) but less FBAPs larger than 2.5 μm (panel f). These results suggest continental aerosols enriched with fine mode aerosols are dominate INP sources during non-dust days and exhibit substantially different size and contribution of INPs in comparison with dust episode days.

Figure 6 generally shows that dust days have much higher number concentration of total FBAPs indicated by Fluo_WIBS>0.5µm, total_ in the morning from 00:00 to 08:00 h. The enriched FABPs are primarily attributed to C_WIBS_ and BC_WIBS_ particles (from 00:00 to 08:00 h in Fig. 6e, h) which are often associated with dust events and biological particles^47^. The enriched C_WIBS_ and BC_WIBS_ may be large-sized (3–10 μm as seen in Fig. 6e from Gao et al.^32^) dust-containing particles that are more effective INP (Fig. S29f) than seen for smaller-sized particles, explaining higher INP concentrations on dust days in the morning (Figs. 2e to 5e) compared with those on non-dust days (Figs. 2d to 5d).

From 08:00 to 16:00 h, Fluo_WIBS>0.5µm, total_ particles on non-dust days majorly consist of ABC_WIBS_, B_WIBS_, and BC_WIBS_ particles, while Fluo_WIBS>0.5µm, total_ particles on dust days mostly include ABC_WIBS_, A_WIBS_, C_WIBS_, and BC_WIBS_ particles (Fig. 6). The higher concentrations of ABC_WIBS_ (by a factor of ~2) and B_WIBS_ particles (by a factor of ~4) on non-dust days may explain their higher number concentrations of INPs (by ~50%) compared with those on dust days, given that ABC_WIBS_ are of the highest probability of being biological particles^32,47^ which are active INPs at warm temperatures. B_WIBS_ particles on non-dust days are likely from continental aerosols smaller than 2.0 μm (see Fig. 6c in Gao et al.^32^) and related to bacteria that may be IN active^53^. Differently, the enriched A_WIBS_ and C_WIBS_ particles on dust days are probably of sizes between 3 and 10 μm (see Fig. 6a, d in Gao et al.^32^), which can be attributed to fungal spores and/or fragmented pollen grains of similar sizes^47,50^ or small bacteria combined with large-sized dust particles^45^. Additionally, the higher concentrations of FABPs for both non-dust and dust days between 08:00 and 16:00 h than the other periods of the day coincide with the noon peaks of INPs for both cases (Figs. 2 to 5). Notably, the peak of INPs on non-dust days at 12:00 h overall shows an overlap with that of ABC_WIBS_ (Figs. 5d and 6b) but not with APS_>2.5μm_ particles (showing a peak at 14:00 h in Fig. 3d), suggesting that the contribution of FBAPs to the observed INPs is more important than that of total coarse-sized ( > 2.5 μm) aerosol particles. In contrast, the coincided overlaps of INPs and APS_>2.5μm_ particles on dust days as shown in Fig. 3e indicate the significant role of coarse-sized dust particles as INP contributors.

From 16:00 h to the end of the day, Fluo_WIBS>0.5µm, total_ particles on non-dust days are up to twofold higher than those for dust days (Fig. 6a). Those enriched FABPs on non-dust days are generally contributed by ABC_WIBS_, B_WIBS_ and BC_WIBS_ particles (Fig. 6b, d, h). In particular, the elevated concentrations of FABPs (ABC_WIBS_, B_WIBS_ and BC_WIBS_) coincide with the spark of INP evening peak (by 50%, from 20:00 to 21:00 in Figs. 2d to 5d) on non-dust days, suggesting the contribution of FBAPs to the increase in INPs. In contrast, a relatively constant FABP median concentration (e.g., Fluo_WIBS>0.5µm, total_ in Fig. 6a and the other types) corresponds to stable INP concentrations on dust days during the same period (Figs. 2e to 5e). Additionally, the increased B_WIBS_ and BC_WIBS_ particles with a peak around 20:00 h on non-dust days may be attributed to the elevated eBC particles (Fig. S14d).

In summary, FBAPs and dust particles are the key drivers for the INP diurnal cycles observed at (HAC)^2^. ABC_WIBS_ FBAPs are the most important particles regulating INPs in the PBL without dust events. Dust events can substantially enrich larger-sized dust containing FABPs (A_WIBS_ and C_WIBS_) during noon time when PBL is high (suggesting that airmasses in PBL may also be of the sources of A_WIBS_ and C_WIBS_ FBAPs), while they supply more large-sized C_WIBS_ and BC_WIBS_ FABPs when PBLH is lower during nighttime, which also contributes to the observed INPs.

### Vertical INP distributions with respect to PBL/FT interface for each atmospheric classification

**Fig. 7:**
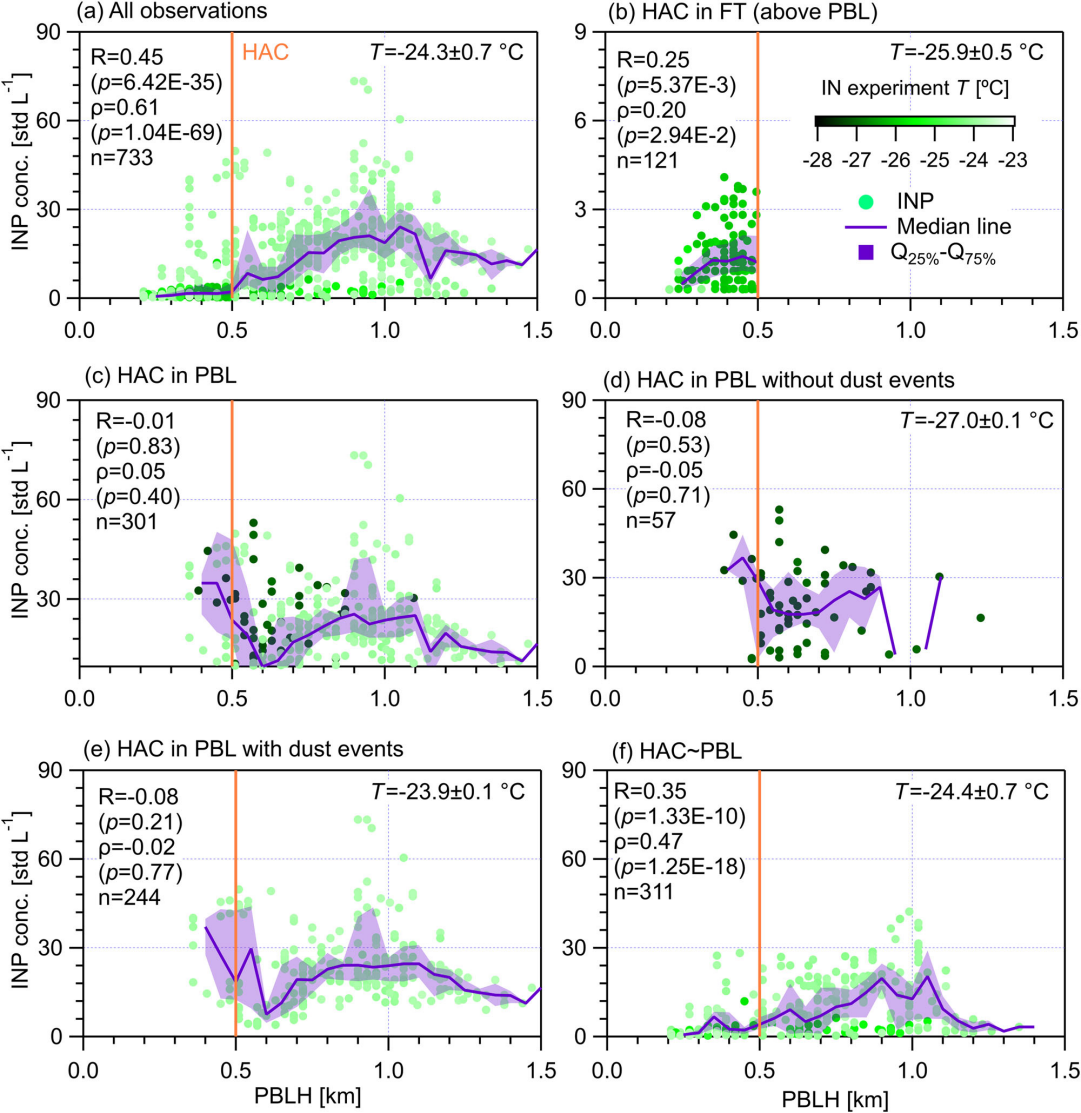
INP concentration distribution as a function of PBLH. The color scale shows the ice nucleation experiment temperature. Solid lines indicate median values and the shading area around the median line shows the range between 25th and 75th quartiles. INP data was filtered within a small temperature range as indicated in each panel, to reduce the temperature dependence of INPs. Different (HAC)^2^ atmospheric conditions are classified in different panels. **a** All observations during the campaign. **b** For days only in the FT. **c** For days only in the PBL. **d** Days in the PBL without dust events. **e** Days in the PBL with dust events. **f** Days not exclusively in the PBL or FT. Data points were resampled for every 20 min. The Pearson correlation coefficient (*R*) and the Spearman rank coefficient (ρ), as well as corresponding *p* values, are provided to evaluate the correlation between INP concentration and PBLH. The *p* value is the probability of obtaining an *R* (ρ) value no smaller than the true *R* (ρ) value if there is no liner correlation between INP concentration and PBLH. The *n* value is the number of data points for the statistical analysis.

Figure 7 illustrates INP concentration distributions with PBLH under different atmospheric conditions. In general, INPs show a positive and significant linear correlation with increasing PBLH (Fig. 7a). For days exclusively in the FT (Fig. 7b), INPs overall decrease with decreasing PBLH, showing a statistically significant (*p* = 5.37 × 10^−3^) but weak correlation (*R* = 0.25). This means that INPs become rarer deeper inside the FT, as the total aerosol particles do (Supplementary Figs. S31b to S36b). For days only in PBL without dust events (Fig. 7d), INPs show small variations with changing PBLH. This is because airmasses in the PBL are intensively mixed^54^ and most aerosol particles in the PBL generally show insignificant dependence on the PBLH (Figs. S31d to S33d), except FBAPs, i.e., Fluo_WIBS>0.5µm, total_ and ABC_WIBS_ (Figs. S34d and S35d). Supplementary Figs. S34d and S35d present that both Fluo_WIBS>0.5µm, total_ and ABC_WIBS_ for non-dust days increase with increasing PBLH, showing strong correlations (*R* = 0.72 and 0.64 respectively). The reason for the different dependences of INPs and FBAPs on the PBLH for the case of non-dust days in the PBL may be twofold. First, considering the active ice nucleation ability of bioaerosols that can activate as ice at warm temperatures (>−20 °C)^39^, FBAPs may only partly contribute to INPs tested at colder temperatures (~−27 °C) for (HAC)^2^ in the PBL. In addition, FBAPs, such as ABC_WIBS_ and Fluo_WIBS_, may not include the total number of particles with biological material that contribute to the observed INPs. For days when (HAC)^2^ is only in the PBL with dust events (Fig. 7e), the INP concentration does not vary significantly with changing PBLH and it shows an INP peak close to PBLH = 1.0 km. The peak can be explained by the peak of APS_>2.5μm_ at the same PBLH level (Supplementary Fig. S33e), given that coarse-sized particles are more effective INPs. For days not exclusively in the PBL or FT (Fig. 7f), the positive correlation between INP and PBLH is moderate and significant, similar to the overall observations for the campaign.

## Discussion and conclusions

We demonstrate the existence of diurnal INPs cycles for moderately-cool mixed-phase orographic clouds (~−25 °C) in the E. Mediterranean and determine their drivers. The diurnal cycles of aerosols sourced from planetary boundary layer (PBL; e.g., bioaerosol) and remotely transported particles (e.g., Saharan dust) regulate the INP periodicity and variations. A strong diurnal INP cycle is seen when the observation site resides in the PBL and practically vanishes when in the free troposphere (FT). In particular, we reveal the relative importance of different INP sources, including bioaerosols, dust, and eBC-containing particles, for the observed INP diurnal cycles. We show that INPs in the FT originate from total aerosol particles in different size ranges larger 0.5 μm (Table 1) but are not correlated with any types of fluorescent biological aerosol particles (FBAPs, Table 3). This highlights the INP population observed in the FT is different from that in the PBL. Likely, this is because INPs in the FT are far-transported and aged particles, whereas INPs in the PBL are much closer to their sources, e.g., local biological particles. This is also indicative of the necessities of further studies on the source apportionment of INPs in the FT, which is already suggested by Gao et al.^32^ and will be investigated in our next field campaign at the (HAC)^2^.

The diurnal cycles of FBAPs in the PBL significantly contribute to the INP cycles at warm MPC temperatures (>−20 °C). Abrupt aerosol source changes caused by dust plume intrusions can perturb the diurnal variability and vertical distribution of INPs. The presence of Saharan dust drives INP variability generally by enriching larger-sized particles (>1.0 μm; Table 1), while in its absence INPs are overall more linked to particles of size between 0.5 and 1.0 μm (Table 1). The elevated concentration of coarse-sized dust particles during Saharan dust events does not significantly increase INPs during daytime because the uplifting of PBL airmasses with increased PBLH may bring more continental aerosol that impedes dust plume intrusions or mixes with dusty aerosols inducing aging and activity suppression of INPs. The competition between uplifting PBL airmasses and dust plume intrusions may also reduce the supply of bioaerosols from the lower PBL. During nighttime with Saharan dust events, coarse-sized dust particles can increase INPs when the availability of PBL airmasses is low because of PBL contraction, which diminishes the variance of INPs throughout the day. Thus, the different contribution of dust particles during daytime and nighttime provides a control test and reveals the important role of bioaerosols for INPs in the MPC regime during daytime. It is also notable that total FBAP concentrations explain more than 90% of INP concentrations during the dust event (Fig. S13h, which shows 97% of INPs are within a factor of 3 compared to Fluo_WIBS>0.5μm, total_). Additionally, we demonstrate that eBC-containing particles (originating from anthropogenic activities or wildfires) play a negligible role in the diurnal periodicity of INPs in the MPC regime.

In conclusion, we show that the diurnal cycles of INPs for orographic clouds in the E. Mediterranean is driven by the PBL airmasses, in which bioaerosols are a major driver of this variability in the absence of dust. The presence of dust influences the variabilities of both FBAPs and INPs, showing enrichment in INPs particularly in the morning when PBL aerosol sources are less available with low PBLHs. Considering that bioaerosols are present at forested mountainous regions, they are one of the key regulators that primarily modulate the ice formation in orographic clouds and may also influence secondary ice production^18^. Such INP sources and diurnal forcing of clouds are rarely considered in models but are expected based on the generality and ubiquity of the sources determined here. Given the importance of bioaerosols demonstrated in this study and the reported importance of correct vertical distribution of INPs for the formation of extreme precipitation in mountainous environments^18^ and potentially for cloud system development^17^, it becomes clear that such sources and corresponding cycles may be underappreciated drivers of cloud formation, aerosol-cloud interactions, and extreme events.

## Methods

### CALISHTO field campaign

To investigate aerosol-cloud interactions (including INP abundance and variability) in the eastern Mediterranean region, a field campaign, called the Cloud-AerosoL InteractionS in the Helmos background TropOsphere (CALISTHO, https://calishto.panacea-ri.gr/)^18,31–33^ was carried out on the basis of the Hellenic Atmospheric Aerosol and Climate Change station (termed (HAC)^2^, ~2.3 km a.s.l., 37.984033° N and 22.196060° E) at Mount Helmos (Greece) between October and November 2021. The (HAC)^2^ is an observation site that allows the study of INPs both under the PBL and FT conditions^55^ because of its high altitude. The instrumentation set-up and timeseries results of different measurements are presented in Supplementary Figs. S1 and S2, respectively. An online INP spectrometer called Portable Ice Nucleation Experiment (PINE)^28^ was used to measure INP concentrations at the (HAC)^2^ from an omnidirectional total inlet with a temporal resolution of 6 ~ 7 min. The PINE inlet has an 80% sampling efficiency for particles between 3 and 5 μm, and it decreases to approximately 50% for particles between 5 and 10 μm. In this study, the PINE was operated in a *T* range from ~−24 to −27 °C and *S*_w_ (saturation ratio with respect to water) >1.0 to measure INPs activating as ice in all freezing modes^32^. A wind Doppler Lidar (HALO, StreamLine Wind Pro model, HALO Photonics), deployed at a lower site than the (HAC)^2^ (by ~0.5 km), was used to measure the PBLH and to determine the (HAC)^2^ position with respect to the PBL-FT interface. Aerosol properties and meteorological parameters at the (HAC)^2^ were monitored simultaneously to understand the variations of observed INPs. The characterized aerosol properties include the number concentration of aerosol particles larger than 95 nm recorded by a scanning mobility particle sizer (SMPS_>95nm and <800nm_, SMPS model 3938, TSI Inc., US, measuring particle size distributions in the size range between 10 to 800 nm), the total concentration of aerosol particles (0.5‒20 μm, aerodynamic diameter) recorded by an aerodynamic particle sizer (APS_>__0__.__5__μ__m__, total_, APS model 3321, TSI Inc., US), the concentration of particles (0.5‒30 μm, optical diameter) showing fluorescence in three fluorescent channels of a wideband integrated bioaerosol sensor (WIBS-5/NEO, Droplet Measurement Technologies, LLC. US), aerosol scattering coefficient at 450 nm (Scatt450nm) and Ångstrӧm exponent at the 450–700 nm wavelength pair measured by a nephelometer (Model 3563, TSI Inc., US), as well as the mass concentration of elemental black carbon (eBC) measured by an aethalometer (AE31, Magee Scientific, US). The recorded meteorological parameters include wind velocity and direction data, relative humidity *wrt*. water (RH_w_) and *T*_ambient_. In addition, dust particle mass concentrations at different altitudes are calculated by the SKIRON model^56,57^ to diagnose the presence of Sahara dust events. The field campaign data analyzed covered more than 6 weeks, from October 12 to November 24, 2021.

### Planetary boundary layer (PBL) condition determination

The HALO wind lidar measures the vertical velocity of air masses carrying micron-sized aerosol particles at a stare mode emitting pulsed laser beams at 1.5 μm. Using the Doppler effect, the radial wind velocity along the direction of the laser beam can be retrieved and the distance between scatters (aerosols) in the beam path can be calculated^58,59^. The maximum detection range varies from 2.0 to 3.0 km depending on the micron-sized aerosol load in the atmosphere^60^. More detailed information about the measurements and calculation methods for PBLH can be found in our parallel work^31,32^ for the CALISHITO campaign. To determine the PBL condition at the (HAC)^2^ for time periods when PBLH is missing due to insufficient micron-sized aerosol load, a SMPS_>95nm, <800 nm_ threshold value of 100 std cm^−3^ was used to diagnose the position of the (HAC)^2^ relative to the PBL-FT interface^27,34^. For SMPS_>95nm, <800nm_ > 100 std cm^−3^, it means the (HAC)^2^ is within the PBL. Otherwise, it may be above the PBL and more in the FT.

### The determination of dust events

To determine the presence of dust events around the (HAC)^2^, the dust mass concentration calculated by the SKIRON model^56,57^ and the Ångstrӧm exponent^32^ at the 450–700 nm wavelength pair (see Supplementary S1) measured by a nephelometer are used^61^. From Supplementary Fig. S2f, dust mass concentration decreases with decreasing altitude below the (HAC)^2^. Thus, the dust mass concentration at the (HAC)^2^ (~2.3 km a.s.l.) may be higher than the concentration calculated by the model at the highest altitude (2.17 km). Also, the Ångstrӧm exponent is compared to a threshold value of 1.0 and a smaller Ångstrӧm exponent value indicates the presence of dust events at the (HAC)^2^. Additionally, the footprints of air masses from Sahara provided by air mass dynamic simulations in our parallel work confirm that dust plumes originate from the Saharan desert.

### Daily based data classification based on (HAC)^2^ condition with respect to the PBL-FT interface

An observation day will be classified as a scenario of (HAC)^2^ only in the PBL (or (HAC)^2^ only in the FT) if the PBLH is larger (or lower) than 0.5 km for more than 23 h throughout the day, which otherwise will be attributed to a day neither exclusively in the FT nor in the PBL (i.e., (HAC)^2^ ~ PBL top). When PBLH data is missing, SMPS_>95nm, <800nm_ results will be used to diagnose the atmospheric condition at (HAC)^2^. Based on the above criteria and considering the influence of dust events, we classify the daily results into four cases as introduced in the main text, to calculate the diurnal cycles of INPs and aerosol properties using 20 min averaged data. The median values and the 25th to 75th percentile ranges (Q_25%_–Q_75%_) are used to describe the INP and aerosol particle concentration levels of the diurnal cycles. Away from sources, atmospheric aerosol particles tend to be log-normally distributed^62^. Assuming the log-normally distributed data without any skewness, the median value equals to the log-normal mean value. Therefore, we report the median results as Brunner et al.^27^.

## Supplementary information

Supplementary Information

## Data Availability

The data presented in this publication are available at 10.16904/envidat.551.
