# Supplementary material for: On the drivers of ice nucleating particle diurnal variability in Eastern Mediterranean clouds
Source: NPJ Clim Atmos Sci. 2025 May 5;8(1):160. doi: 10.1038/s41612-024-00817-9 (PMC12052592; doi:10.1038/s41612-024-00817-9)
Supplement: Supplementary file 1 — Supplementary Information [file 41612_2024_817_MOESM1_ESM.docx]

*Supplement of*

**On the drivers of ice nucleating particle diurnal variability in Eastern Mediterranean clouds**

Kunfeng Gao et al.

*Correspondence to*: Athanasios Nenes ([athanasios.nenes@epfl.ch](mailto:athanasios.nenes@epfl.ch)) and Kunfeng Gao ([kunfeng.gao@epfl.ch](mailto:kunfeng.gao@epfl.ch))

# S1 Observation location and instrumentation setup

CALISHITO campaign took place at Mt. Helmos in Peloponnese, Greece (eastern Mediterranean). A synergy of measurement techniques was used to investigate the aerosol-cloud interaction processes, including in-situ aerosol property measurements, remote sensing measurements and modeling experiments (Fig. S1). In-situ measurements were performed at the high altitude Helmos Hellenic Atmospheric Aerosol and Climate Change station (termed (HAC)^2^, ~2.3 km above sea level (a.s.l.), 37.984033° N and 22.196060° E). An ice nucleating particle (INP) spectrometer called portable ice nucleation experiment (PINE)^1^ was used to measure INPs at (HAC)^2^ in the temperature range between −23 and −28 °C under water saturated conditions (saturation ratio with respect to water, *S*_w_>1.0) for every 6~7 minutes. PINE inlet has a 80% sampling efficiency for particles between 3 and 5 μm, and it decreases to approximately 50% for particles between 5 and 10 μm. In-situ aerosol particle size distribution was measured by a scanning mobility particle sizer spectrometer (SMPS; Vienna-type differential mobility analyzer and condensation particle counter 3772, TSI Inc., US) from 10 to 800 nm (electric mobility diameter) and by an aerodynamic particle sizer spectrometer (APS; model 3321, TSI Inc., US) from 0.5 to 20 μm (aerodynamic diameter). Fluorescent particle concentrations were monitored by a wideband integrated bioaerosol sensor-New Electronics Option (WIBS-5/NEO, Droplet Measurement Technologies, LLC. US) in the size range between 0.5 and 30 μm (optical diameter), including Fluo_WIBS_ showing fluorescence in any one of the three fluorescent channels and ABC_WIBS_ showing fluorescence in all three fluorescent channels^2^. A nephelometer (Model 3563, TSI Inc., US) was used to measure the light scattering coefficients at 3 wavelengths (Scatt450nm, Scatt550nm and Scatt700nm). The Ångstrӧm exponent (α) was calculated using $\alpha=-\frac{ln[{{Scatt}_{700 nm}}/{{Scatt}_{450 nm}}]}{ln({700}/{450})}$^3^. The mass concentration of aerosol particles containing elemental black carbon (eBC) was monitored by an aethalometer (AE31, Magee Scientific, US) with a time resolution of 2 min. A Doppler wind lidar (StreamLine Wind Pro model, HALO Photonics, UK), deployed at Vathia Lakka (VL, 0.5 km lower than (HAC)^2^), was used to detect the planetary boundary layer height (PBLH) by measuring the vertical velocity distribution of air masses. The position where the standard deviation of air mass vertical velocity is smaller than 0.1 m^2^ s^-2^ is determined as outside of the PBL, i.e., the bottom of the free troposphere (FT)^4^. The SKIRON model^5-6^ was used to calculate the dust mass concentration close to (HAC)^2^ at different altitudes of 1250, 1614, 1881 and 2170 m a.s.l. Additionally, the meteorological parameters at (HAC)^2^, such as ambient temperature (*T*_ambient_), relative humidity with respect to water (RH_w_), horizontal wind velocity and direction, were also recorded.


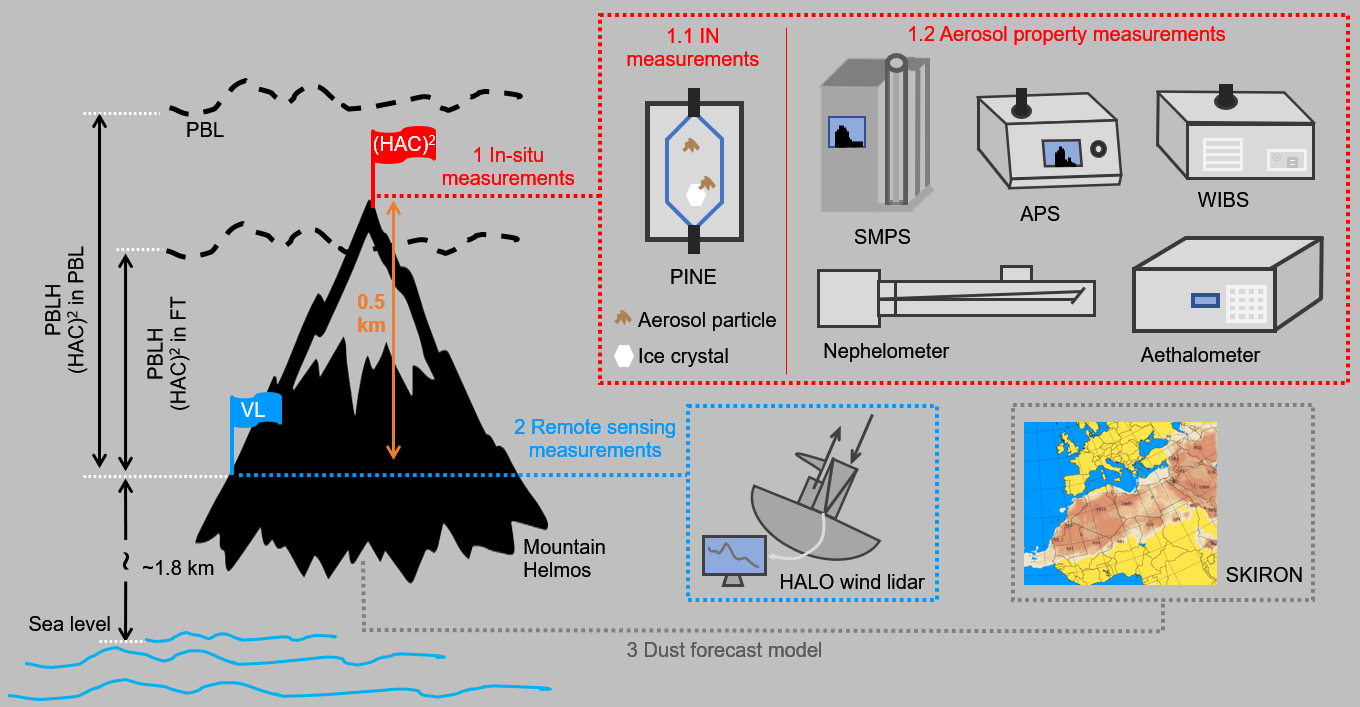


Fig. S1. Illustration of measurement sites and instrumental setup for CALISHITO campaign at Mt. Helmos. 1 (HAC)^2^ represents the High altitude Helmos Hellenic Atmospheric Aerosol and Climate Change station at 2314 m a.s.l. where in-situ measurements were performed, including ice nucleation (IN) measurements and aerosol property measurements. The ambient aerosol flow was sampled for portable ice nucleation experiment (PINE), scanning mobility particle sizer (SMPS), aerodynamic particle sizer (APS), wideband integrated bioaerosol sensor (WIBS), nephelometer and aethalometer. 2 VL represents Vathia Lakka at an altitude of ~1.8 km, i.e. 0.5 km lower than (HAC)^2^, at which HALO wind lidar was deployed to measure planetary boundary layer height (PBLH). 3 SKIRON is the dust forecast model developed by National and Kapodistrian University of Athens. PBL is planetary boundary layer and PBLH stands for PBL height with respect to the VL location.

# S2 Overview of timeseries results

Figure S2 provides the timeseries of in-situ measurement results. PBLH above VL and the number concentration of aerosol particles between 95 nm and 800 nm recorded by SMPS (SMPS_95nm, <800nm_) are presented in Fig. S2a with a time resolution of 20 min. Hourly-averaged INP concentrations are provided in Fig. S2b with one standard deviation indicated by the shading area. The temperature for INP measurement scales to the color bar. Note, INP concentrations with a time resolution of 20 min is used for INP diurnal cycle calculations in the main text. Figure S2c shows the concentration of aerosol particles with a time resolution of 20 min, including the total particle concentration recorded by both SMPS and APS (SMPS+APS_total_), the total particle concentration recorded by APS (APS_>0.5μm, total_), the concentration of particles larger than 2.5 μm recorded by APS (APS_>2.5μm_), as well as Fluo_WIBS>0.5μm, total_ and ABC_WIBS_ particle concentrations. The SMPS+APS_total_ concentration is superimposed from the SMPS and APS data using the method introduced in Khlystov et al^7^. Figure. S2d plots the timeseries of hourly-averaged Scatt450nm and Ångstrӧm exponent measured by the nephelometer and Fig. S2e shows the timeseries of eBC mass concentration recorded by the aethalometer. Figure S2f shows the extrapolated dust mass concentration vertical profile using the mass concentration of dust particles calculated by SKIRON model at different height levels of 1250, 1614, 1881 and 2170 m a.s.l. (meters above sea level). According to Spyrou et al.^5^, long-range transported dust particles in the SKRION model have a size distribution following a lognormal form with mass median diameter equal to 2.524 μm and geometric standard deviation equal to σ = 2. Dust event days are marked in the panel. The hourly-averaged wind velocity and speed timeseries are provided in Fig. S2g, and hourly-averaged *T*_ambient_ and RH_w_ are shown in Fig. S2h. In addition, different PBL conditions are indicated by the shading area in each panel for days exclusively in the PBL or FT.





Fig. S2. Timeseries of measurement results from October 12 to November 24, 2019. (a) PBLH measured by HALO wind lidar and concentration of particles larger than 95 nm measured by SMPS (SMPS_>95nm, <800nm_). (b) INP concentration observed by PINE. The round marker stands for hourly mean INP concentration values and the temperature condition for IN experiments scales to the color bar. The line and the green shading area are for 3-hour mean values and one standard deviation, respectively. (c) The total aerosol particle concentration recorded by both SMPS and APS (<20 μm, SMPS+APS_total_), the total aerosol particle concentration recorded by APS (0.5−20 μm, APS_>0.5μm, total_), total aerosol particle concentration larger than 2.5 μm recorded by APS (APS_>2.5μm_) and fluorescent particle concentration monitored by WIBS (Fluo_WIBS>0.5μm, total_), as well as concentration of particles showing fluoresce in all three WIBS channels (ABC_WIBS_). (d) Scattering coefficient at 450 nm (Scatt450nm) and Ångstrӧm exponent at the 450-700 wavelength pair observed by nephelometer. The upper limit and lower limit of the scattering coefficient are for 84^th^ and 16^th^ percentiles, respectively. (e) The eBC mass concentration with one standard deviation measured by aethalometer. (f) Vertical dust mass concentration distribution calculated by using SKIRON dust forecast model. The results were extrapolated by using the mass concentration of dust particles calculated by SKIRON model at different height levels (at 1250, 1614, 1881 and 2170 m a.s.l.). The dashed red box indicates an intensive dust event from November 4 to 10. (g) Wind velocity and direction with one standard deviation monitored at the meteorological station at (HAC)^2^. (h) Temperature and RH_w_ with one standard deviation monitored at the (HAC)^2^ meteorological station. The shading area in each panel indicates those calendar days on which (HAC)^2^ is only in the free troposphere (FT) or in the planetary boundary layer (PBL) throughout the day. All the particle concentration values were converted into standard temperature and pressure condition. Any gaps reflect missing data.

# S3 The drivers of PBLH

The PBLH over mountainous terrain is a result of the interaction of the turbulent fluid flows over the complex mountainous topography^8-9^. The ambient temperature (*T*_ambient_) and development of air flows, as well as wind systems, are often thermally driven, which is ultimately regulated by solar radiation^8,10^. Figure S3 compares the overall daily PBLH pattern (all data during the campaign) to the diurnal cycles of *T*_ambient_, horizontal wind velocity and direction observed at (HAC)^2^. The PBLH exhibits a weak diurnal cycle showing the minimum in the morning (around 9:00 AM) and two peaks in the early morning and in the afternoon (Fig. S3a). In general, PBLH shows slightly higher values during daytime, particularly in the afternoon, compared to nighttime. The increasing PBLH since sunrise may result from increasing solar radiation (up to local noon hours) which expands the air volume close to the earth surface and leads to more intensive convection and promoted PBL extension. On the contrary, the decreasing PBLH after sunset is attributed to radiative cooling. The above interpretation is supported by the overall coincident diurnal cycles of PBLH with *T*_ambient_ (Fig. S3a), showing a significant Pearson correlation coefficient (R) of 0.56 (*p*<0.05) and suggesting PBLH is thermally driven. Moreover, the coincidence between PBLH and *T*_ambient_ diurnal cycles is more pronounced and significant for days exclusively in the PBL (Fig. S4c). Not surprisingly, *T*_ambient_ at (HAC)^2^ does not influence the PBLH when the PBL top is below the (HAC)^2^ throughout the day (Fig. S4b).

Figure S3b also shows a negative and significant (*p*<0.05) correlation between PBLH and horizontal wind velocity. The negative correlation can only be seen when PBL top is above the (HAC)^2^ (Fig. S5c to f), possibly a result of the Bernoulli effect on a wind system at a mountain top, which speeds up winds over the mountain top and decreases pressure^9,11^. Thus, air masses over the mountain close to the PBL top can come down and result in decreased PBLH. Also, the non-negative and insignificant effects of horizontal wind velocity on PBLH for the case of (HAC)^2^ in FT provides a negative control for the Bernoulli effect hypothesis (Fig. S5b).

In addition, the negligible linear correlation between PBLH and wind direction (Fig. S3c and Fig. S6) suggests that air streams close to the terrain surface does not influence PBLH over the (HAC)^2^. This aspect may differ from site to site; e.g., Wieder et al.^12^ and Georgakaki et al.^13^ found air streams from the valley may influence the vertical mixing of airmasses close to a mountaintop in the Alps. This may be related to the degree of influences of the site from lower altitudes, or the regional topography and its impacts on turbulence generation^14^. In their mountaintop site classification study, Collaud Coen et al.^14^ reported that (HAC)^2^ receives less influences from surface air masses compared to the Jungfraujoch site in the Alps (3.580 km a.s.l.), which is even higher than the peak investigated by Wieder et al.^12^ and Georgakaki et al.^13^; likely, because the topography in the Alps has many very high peaks in close proximity, while (HAC)^2^ tends to be the dominant (single) peak in the region.

Dust intrusions may perturb the PBLH, being in general associated with higher values during dust days than that of non-dust days (Fig. S4d and e). During dust events at nighttime, the PBLH is 200m higher than in days without dust events. This is partly because the transported dust plumes may interact with solar radiation and generate a temperature inversion higher up, or warm the PBL airmasses because Saharan dust plumes are associated with warmer temperatures overall (by ~8 °C; Fig. S4d and e). The impacts of Saharan dust events on PBLH seen during CALISHTO contrasts the results reported by Gini et al.^15^ who compared the Saharan dust event effects on the PBLH measured at the foot of Mt. Hymettus (Greece), so these effects may be seasonal or depend on the orographic terrain of the site.

The association of SMPS_>95nm, <800nm_ with *T*_ambient_, horizontal wind velocity and direction (not shown) generally shows similar results to those of PBLH, which supports the usage of SMPS_>95nm, <800nm_ as a proxy for being inside or outside the PBL.





Fig. S3. The dependence of PBLH and SMPS *N*_>95nm_ diurnal cycles on meteorological parameters *T*_ambient_, horizontal wind velocity and direction at (HAC)^2^. Diurnal cycles of PBLH and (a) *T*_ambient_, (b) horizontal wind velocity, (c) horizontal wind direction. Diurnal cycles of SMPS_>95nm, <800nm_ and (d) *T*_ambient_, (e) horizontal wind velocity, and (f) horizontal wind direction. Solid lines indicate the median value and the shading area around the median line shows the range between 25^th^ and 75^th^ quartiles. Hourly-averaged data from October 12 to November 24, 2021 is presented and each panel shows a period cycle of 24 h starting at 00:00 UTC+2 (local time) of the day. The Pearson correlation coefficient (*R*) and the Spearman rank coefficient (ρ), as well as corresponding *p* values, are provided to evaluate the correlation between two diurnal cycles in each panel. The *p* value is the probability of obtaining an *R* (ρ) value no smaller than the true *R* (ρ) value if there is no liner correlation between the two parameters. The number of data points (*n*) for each case of above statistical analysis is 24.


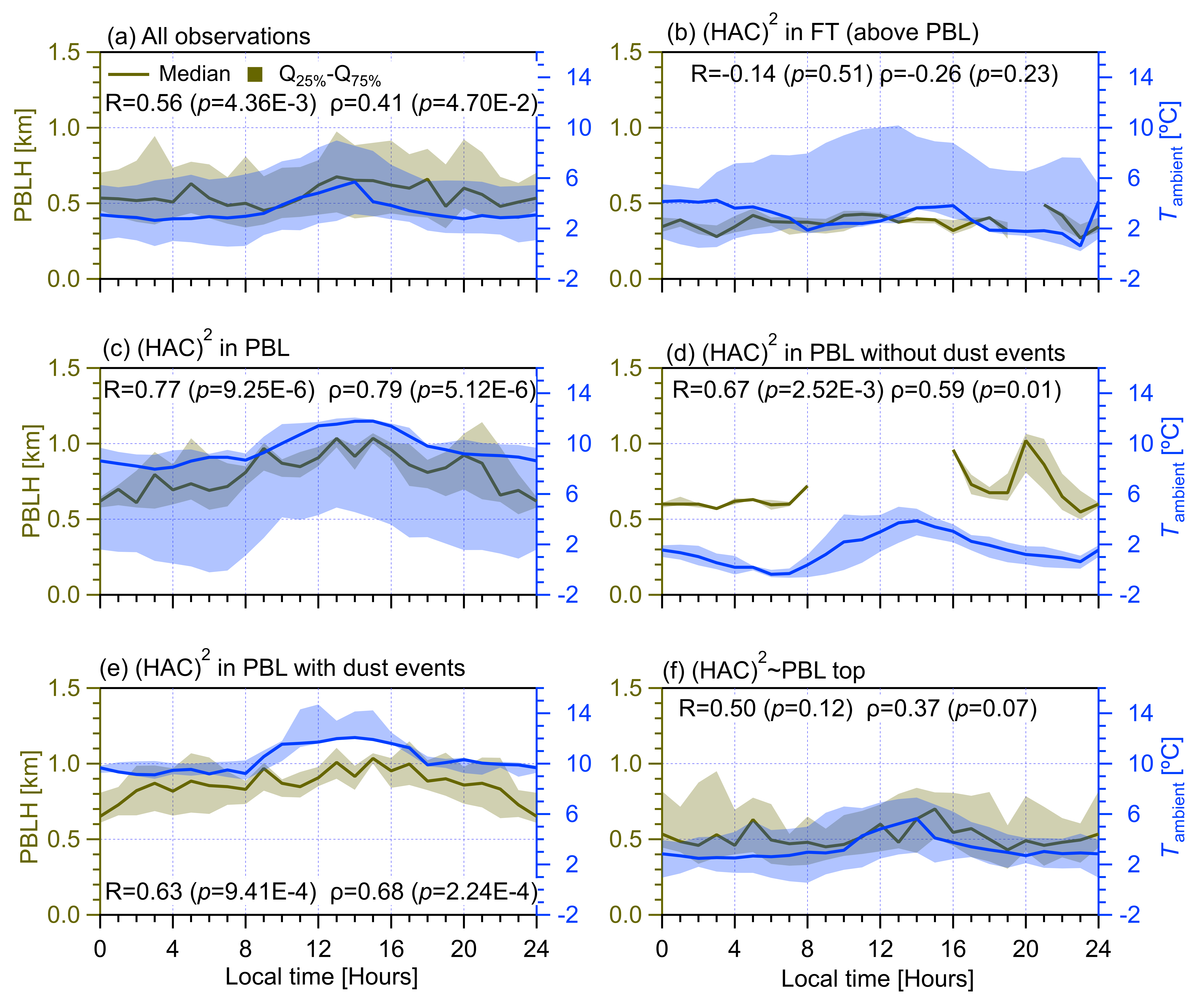


Fig. S4. The dependence of PBLH diurnal cycles on *T*_ambient_ under different atmospheric conditions. Solid lines indicate the median value and the shading area around the median line shows the range between 25^th^ and 75^th^ quartiles. Different (HAC)^2^ atmospheric conditions are classified in different panels. (a) All observations during the campaign. (b) For days only in the FT. (c) For days only in the PBL. (d) Days in the PBL without dust events. (e) Days in the PBL with dust events. (f) Observations for days not exclusively in the PBL or FT. The data points of each (HAC)^2^ position scenario are hourly-averaged and each panel shows a cycle period of 24 h starting at 00:00 UTC+2 (local time) of the day. The Pearson correlation coefficient (*R*) and Spearman’s rank coefficient (ρ), as well as corresponding *p* values, are provided to evaluate the correlation between INP concentration and *T*_ambient_. The *p* value is the probability of obtaining an *R* (ρ) value no smaller than the true *R* (ρ) value if there is no liner correlation between INP and *T*_ambient_. The number of data points (n) for each case of above statistical analysis is 24.


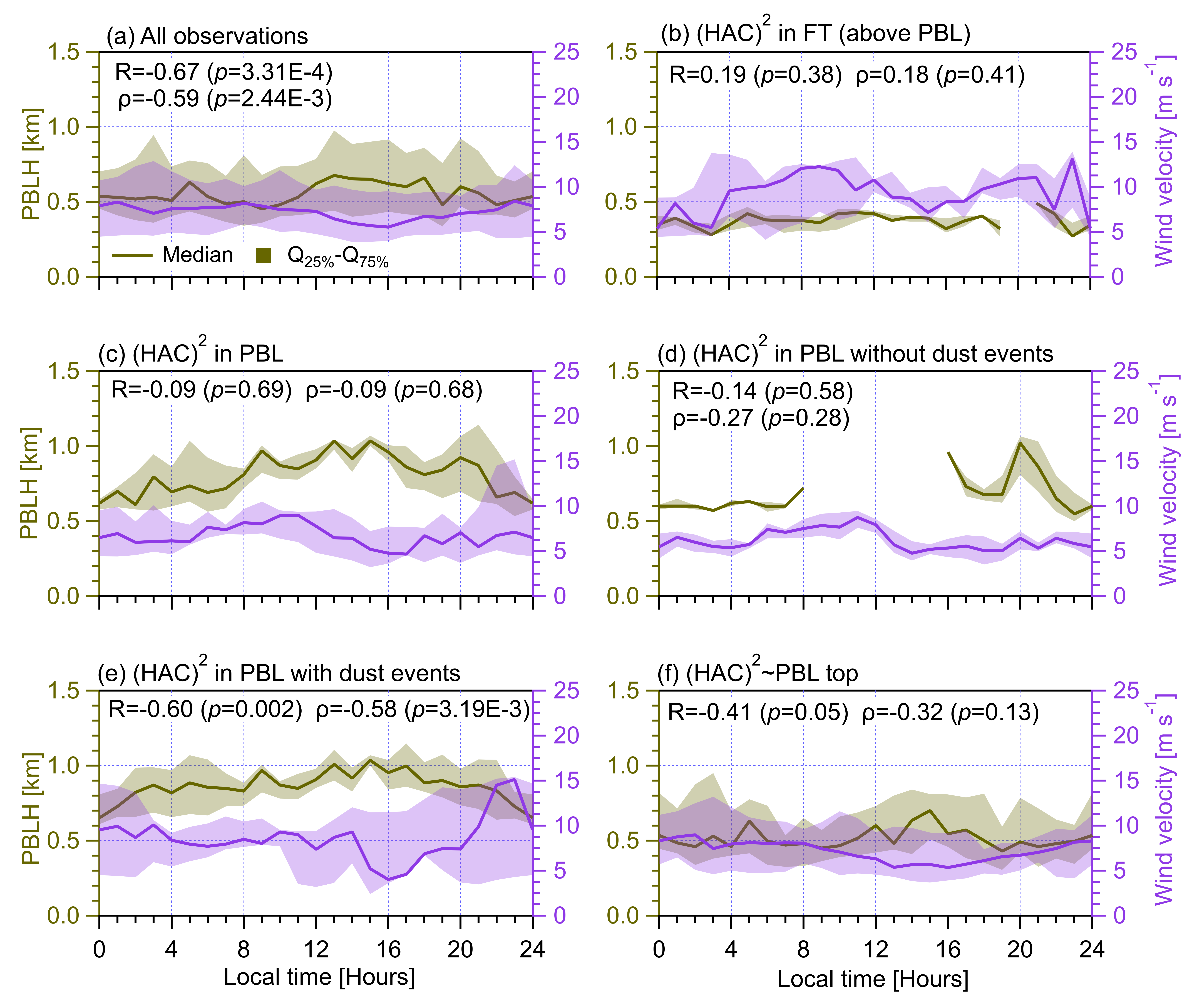


Fig. S5. The same as Fig. S4 but for horizontal wind velocity.


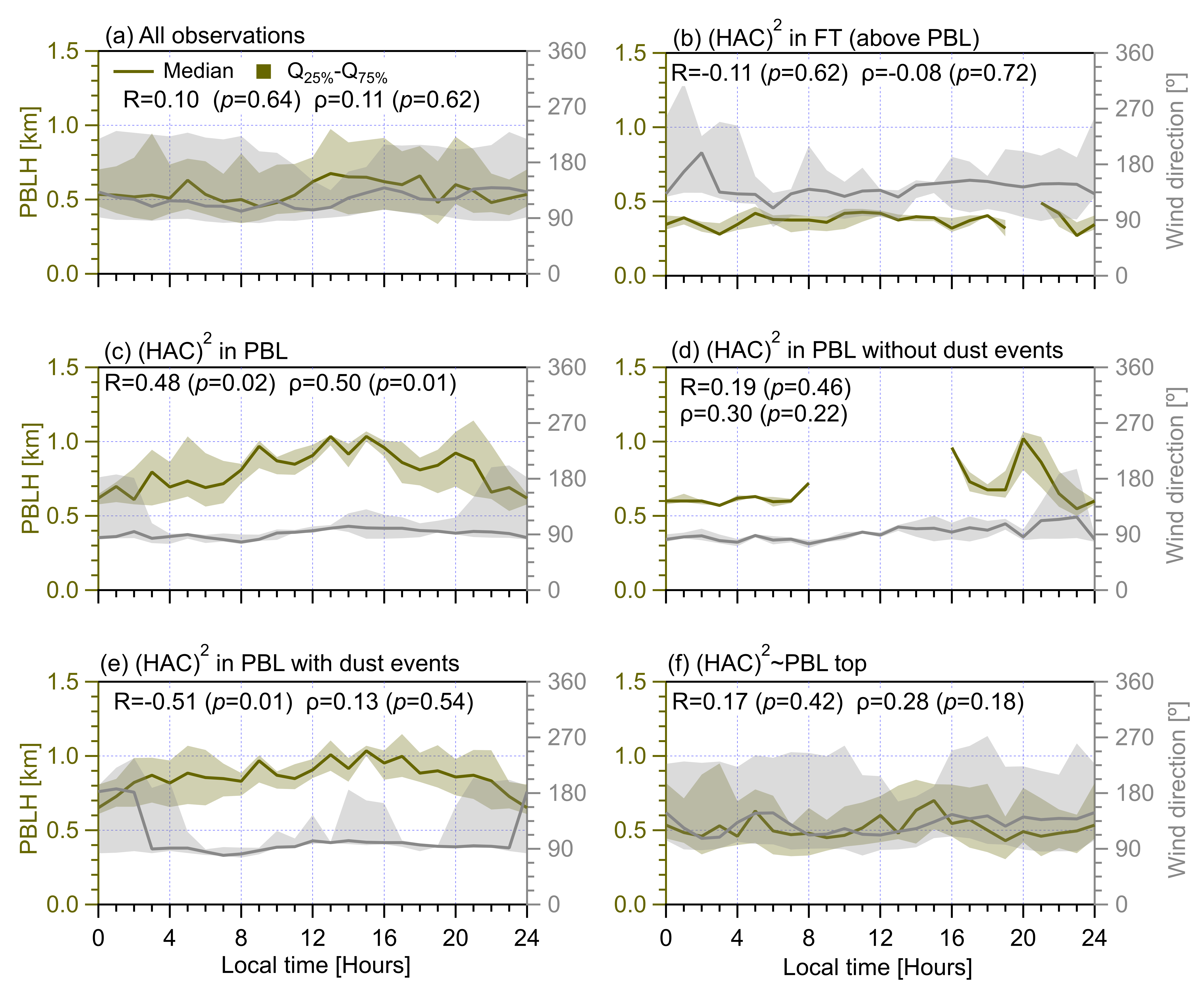


Fig. S6. The same as Fig. S4 but for horizontal wind direction.

S4 The dependence of INP diurnal cycles on aerosol particles with different sizes

This section shows the dependence of INP diurnal cycles on aerosol particles with different sizes measured by both SMPS and APS. It compares the diurnal cycles of INP concentrations with the diurnal cycles of the number concentration of aerosol particles in different size ranges, including SMPS+APS_total_ (Fig. S7), SMPS_<500nm_ (Fig. S8), APS_>0.5μm, <1.0μm_ (Fig. S9), APS_>1.0μm, <1.5μm_ (Fig. S10), APS_>1.5μm, <2.0μm_ (Fig. S11) and APS_>2.0μm, <2.5μm_ (Fig. S12).





Fig. S7. Diurnal cycles of INP (on the left axis) and SMPS+APS_total_ (on the right axis) measured at (HAC)^2^ under different atmospheric conditions. Solid lines indicate the median value and the shading area around the median line shows the range between 25^th^ and 75^th^ quartiles. Different (HAC)^2^ atmospheric conditions are classified in different panels. (a) All observations during the campaign. (b) For days only in the FT. (c) For days only in the PBL. (d) Days in the PBL without dust events. (e) Days in the PBL with dust events. (f) Observations for days not exclusively in the PBL or FT. The data points of each (HAC)^2^ position scenario are hourly-averaged and each panel shows a cycle period of 24 h starting at 00:00 UTC+2 (local time) of the day. The Pearson correlation coefficient (*R*) and Spearman’s rank coefficient (ρ), as well as corresponding *p* values, are provided to evaluate the correlation between INP concentration and SMPS+APS_total_. The *p* value is the probability of obtaining an *R* (ρ) value no smaller than the true *R* (ρ) value if there is no liner correlation between INP and SMPS+APS_total_. The number of data points (n) for each case of above statistical analysis is 24.





Fig. S8. The same as Fig. S7 but the right axis is for SMPS_<500nm_.


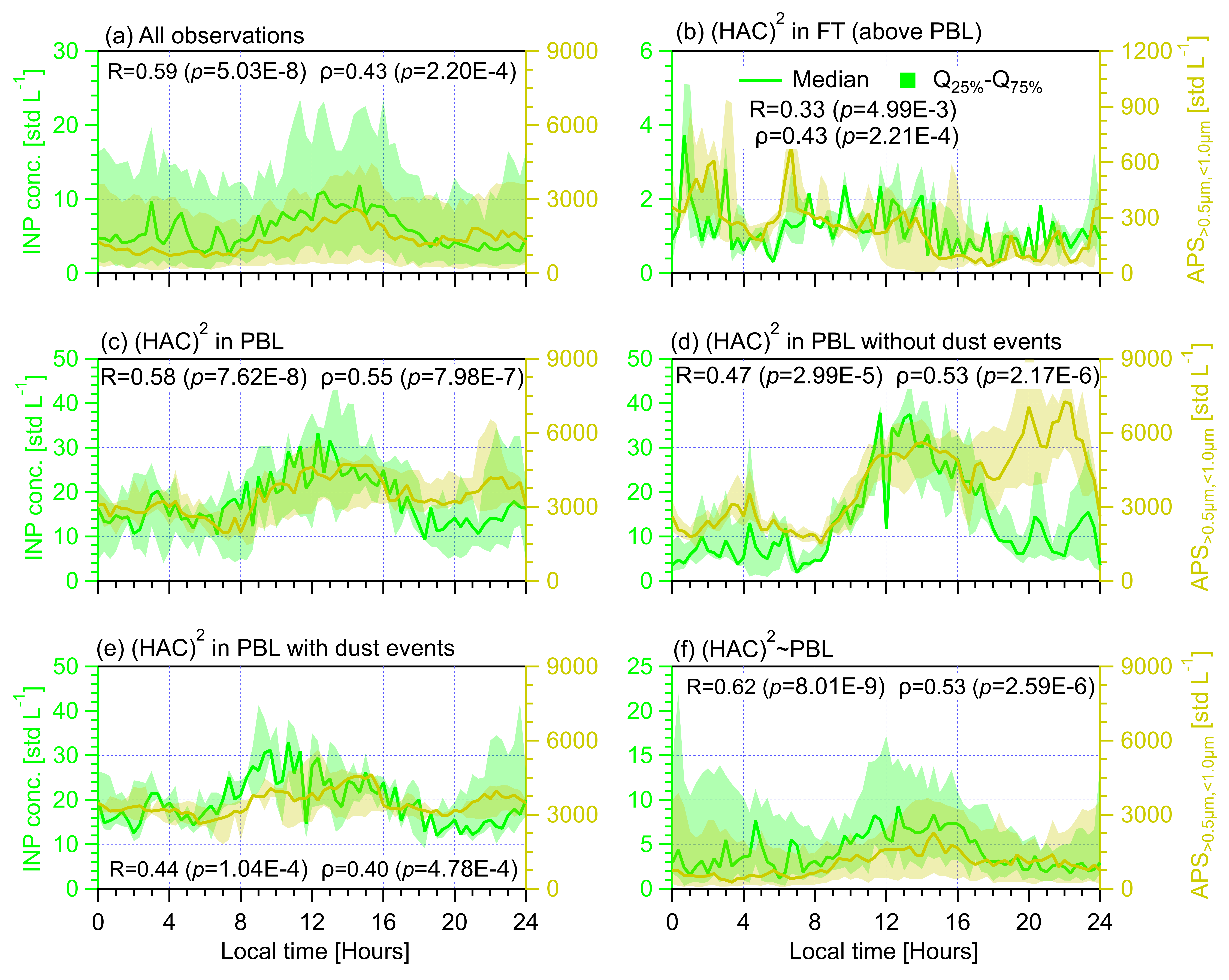


Fig. S9. The same as Fig. S7 but the right axis is for APS_>0.5μm, <1.0μm_.





Fig. S10. The same as Fig. S7 but the right axis is for APS_>1.0μm, <1.5μm_.





Fig. S11. The same as Fig. S7 but the right axis is for APS_>1.5μm, <2.0μm_.





Fig. S12. The same as Fig. S7 but the right axis is for APS_>2.0μm, <2.5μm_.

# S5 The dependence of INP concentration on meteorological conditions and aerosol properties under different PBL conditions

Figure S13 provides results about the dependence of INPs on meteorological parameters and different aerosol properties, by showing the scatter plots of PBLH as a function of *T*_ambient_ (a), RH_W_ (b), wind velocity (c) and wind direction (d), as well as SMPS+APS_total_ (e), APS_>0.5μm, total_ (f), APS_>2.5μm_ (g), Fluo_WIBS_ (h), ABC_WIBS_ (i), Scatt450nm (j), Ångstrӧm exponent (k), eBC (l). Figure S13a shows that *T*_ambient_ is positively and significantly correlated with INPs at (HAC)^2^ in the PBL without the presence of dust events. Such a correlation underlies the dependence of biological emissions on *T*_ambient_. For example, ABC_WIBS_, which is demonstrated as important INP source particles, shows a strong correlation coefficient R=0.88 (*p*=1.88×10^−8^) with *T*_ambient_ on non-dust days. This is consistent with Sjogren et al.^16^who reported a positive of correlation between fluorescent biological aerosol particles and *T*_ambient_ for all seasons in Norway. Figure S13b show that RH_w_ has a negative and significant correlation with INPs on non-dust days in the PBL. This is because high RH_w_ on dust-days are generally during precipitation events which may lead to wet deposition of coarse particles, thus decreasing INP sources. In addition, we observe that a higher wind velocity leads higher INP number concentrations at (HAC)^2^ during dust events, which shows moderate (R=0.55) and significant correlations. The correlation between wind direction and INPs is weak.





Fig. S13. Scatter plots of INPs and different synoptic parameters, and different aerosol properties under different PBL conditions. Different colors are used to indicate different PBL conditions, including days only in the FT, days only in the PBL without and with dust events, as well as days not exclusively in the PBL or FT. Different synoptic parameters and aerosol properties are presented on the *x*-axis of each panel. (a) Ambient temperature (*T*_ambient_). (b) RH_w_. (c) Horizontal wind velocity. (d) Horizontal wind direction. (e) SMPS+APS_total_ particle concentration measured by both SMPS and APS. (f) APS_>0.5μm, total_ particle concentration measured by APS. (g) APS_>2.5μm_ particle concentration measured by APS. (h) Fluorescent particle (Fluo_WIBS>0.5μm, total_) concentration measured by WIBS in any one of the three fluorescent channels. (i) Fluorescent particle (ABC_WIBS_) concentration measured by WIBS in all three fluorescent channels. (j) Light scattering coefficient at 450 nm wavelength (Scatt450nm) monitored by nephelometer. (k) Ångstrӧm exponent derived from the 450−700 nm wavelength pair. (l) The eBC mass concentration measured by an aethalometer. Data points represent hourly-averaged observation value. The Pearson correlation coefficient (*R*) and Spearman’s rank coefficient (ρ), as well as corresponding *p* values, are provided to evaluate the correlation between INP concentration and different parameters. The *p* value is the probability of obtaining an *R* (ρ) value no smaller than the true *R* (ρ) value if there is no liner correlation between INP and the parameter. The *f*_3_ values in panel (h) and (i) stand for the fraction of data points within a factor of 3. The *n* value is the number of data points for the statistical analysis.

# S6 The dependence of INP diurnal cycles on eBC concentration and aerosol optical properties

To support the discussion on the INP diurnal periodicity (Figs. 2 and 3 in the main text), this section shows additional results about the dependence of INPs on eBC concentration and aerosol optical properties, by comparing the diurnal cycles of INPs with the diurnal cycles of the median values of eBC (Fig. S14), Scatt450nm (Fig. S15) and Ångstrӧm exponent (Fig. S16).





Fig. S14. The same as Fig. S7 but the right axis is for hourly-averaged eBC.





Fig. S15. The same as Fig. S7 but the right axis is for hourly averaged Scatt450nm.





Fig. S16. The same as Fig. S7 but the right axis is for hourly averaged Ångstrӧm exponent.

# S7 The dependence of INP diurnal cycles on fluorescent biological aerosol particles with different sizes

This section shows the dependence of INP diurnal cycles on fluorescent biological aerosol particles (FBAPs) with different sizes measured by WIBS. It compares the diurnal cycles of INP concentrations with the diurnal cycles of the number concentration of fluorescent biological aerosol particles in different size ranges, including Fluo_WIBS>0.5μm, <1.0μm_ (Fig. S17), Fluo_WIBS>1.0μm, <1.5μm_ (Fig. S18), Fluo_WIBS>1.5μm, <2.0μm_ (Fig. S19), Fluo_WIBS>2.0μm, <2.5μm_ (Fig. S20) and Fluo_WIBS>2.5μm_ (Fig. S21).





Fig. S17. The same as Fig. S7 but the right axis is for Fluo_WIBS>0.5μm, <1.0μm_.





Fig. S18. The same as Fig. S7 but the right axis is for Fluo_WIBS>1.0μm, <1.5μm_.


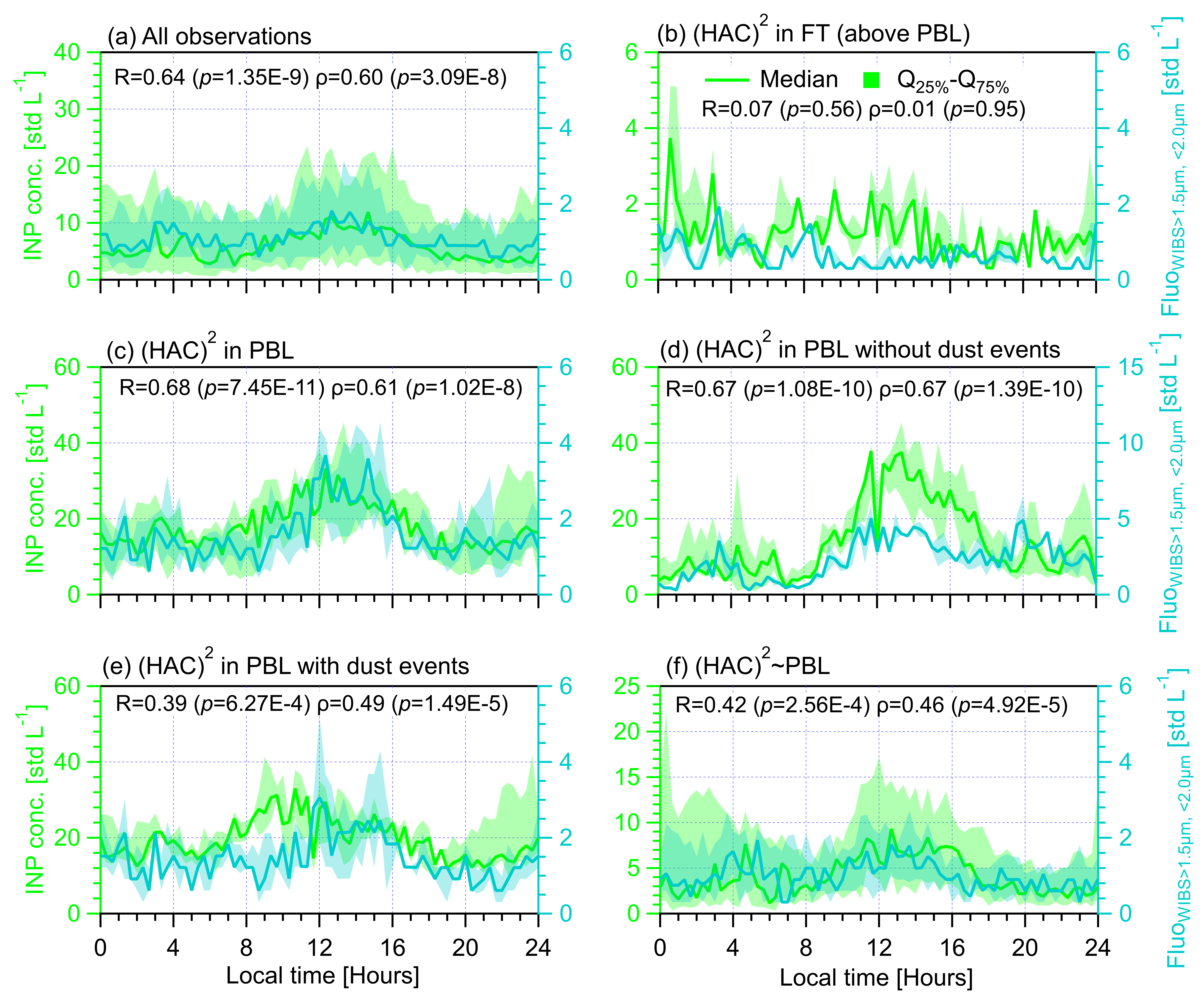


Fig. S19. The same as Fig. S7 but the right axis is for Fluo_WIBS>1.5μm, <2.0μm_.


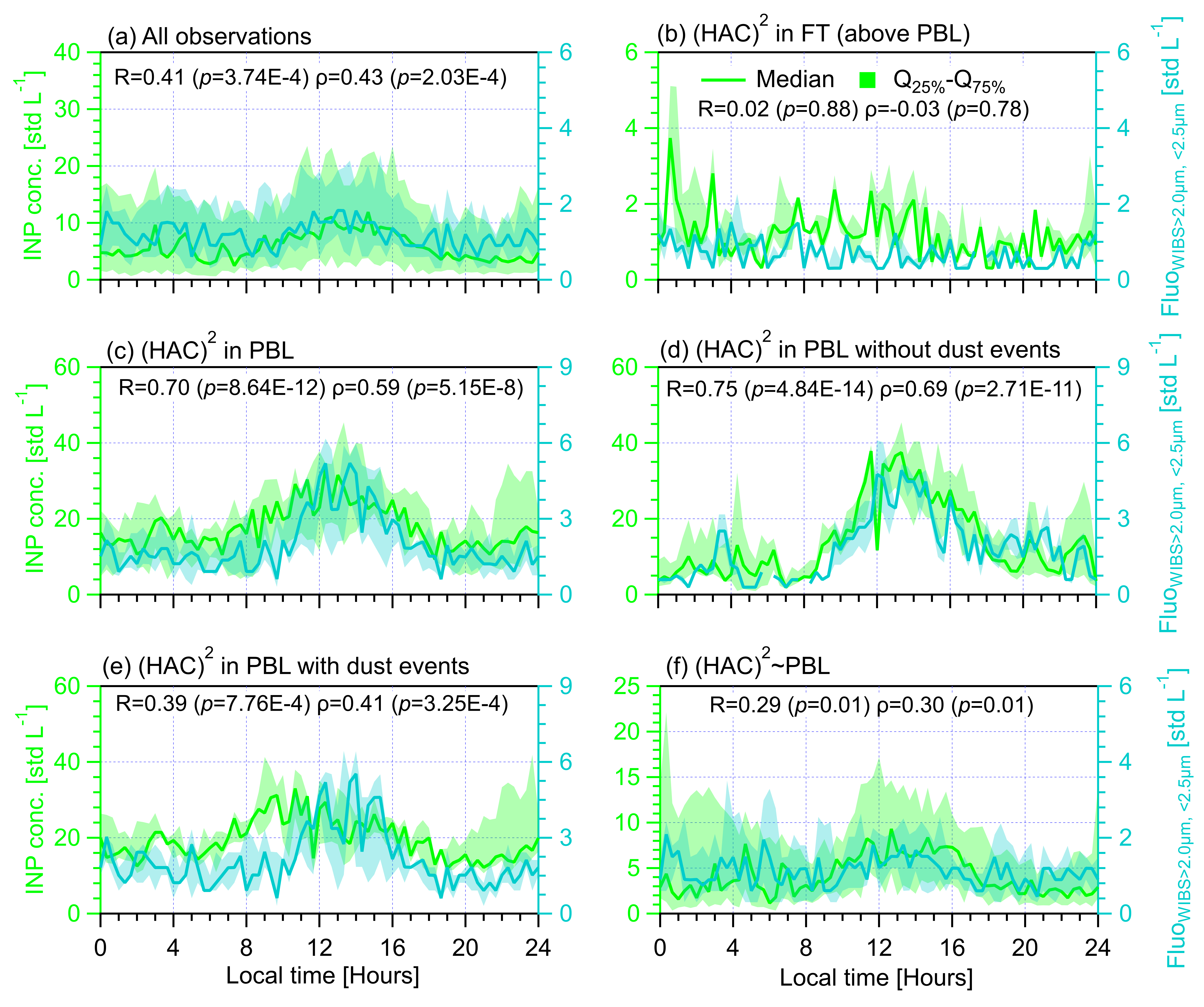


Fig. S20. The same as Fig. S7 but the right axis is for Fluo_WIBS>2.0μm, <2.5μm_.





Fig. S21. The same as Fig. S7 but the right axis is for Fluo_WIBS>2.5μm_.

## S8 The dependence of INP diurnal cycles on different types of fluorescent biological aerosol particles

This section shows the dependence of INP diurnal cycles on different types of fluorescent biological aerosol particles (FBAPs) measured by WIBS. It compares the diurnal cycles of INP concentrations with the diurnal cycles of the number concentration of different types of fluorescent biological aerosol particles, including A_WIBS_ (Fig. S22), B_WIBS_ (Fig. S23), C_WIBS_ (Fig. S24), AB_WIBS_ (Fig. S25), AC_WIBS_ (Fig. S26) and BC_WIBS_ (Fig. S27).





Fig. S22. The same as Fig. S7 but the right axis is for A_WIBS_.


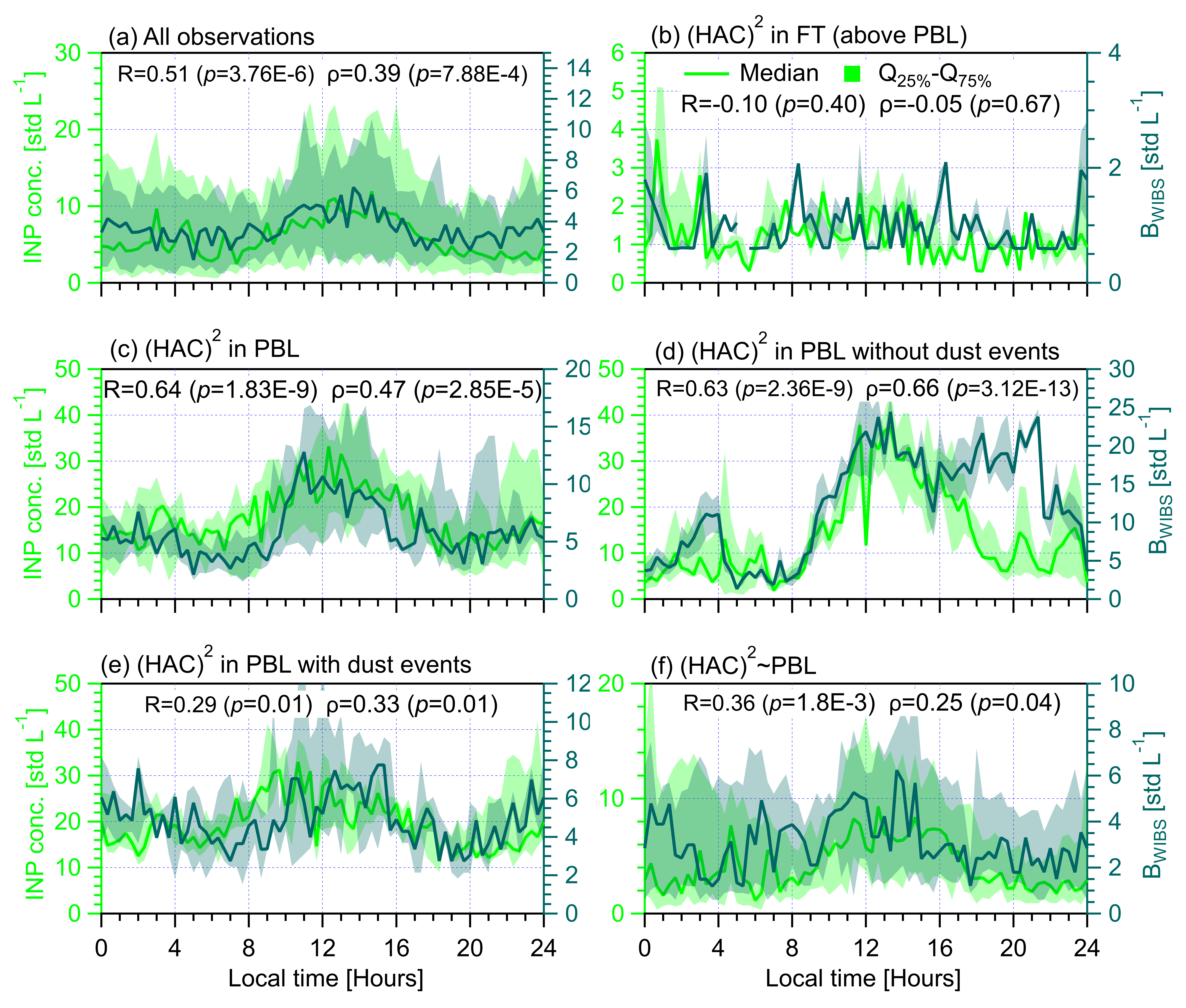


Fig. S23. The same as Fig. S7 but the right axis is for B_WIBS_.





Fig. S24. The same as Fig. S7 but the right axis is for C_WIBS_.





Fig. S25. The same as Fig. S7 but the right axis is for AB_WIBS_.





Fig. S26. The same as Fig. S7 but the right axis is for AC_WIBS_.


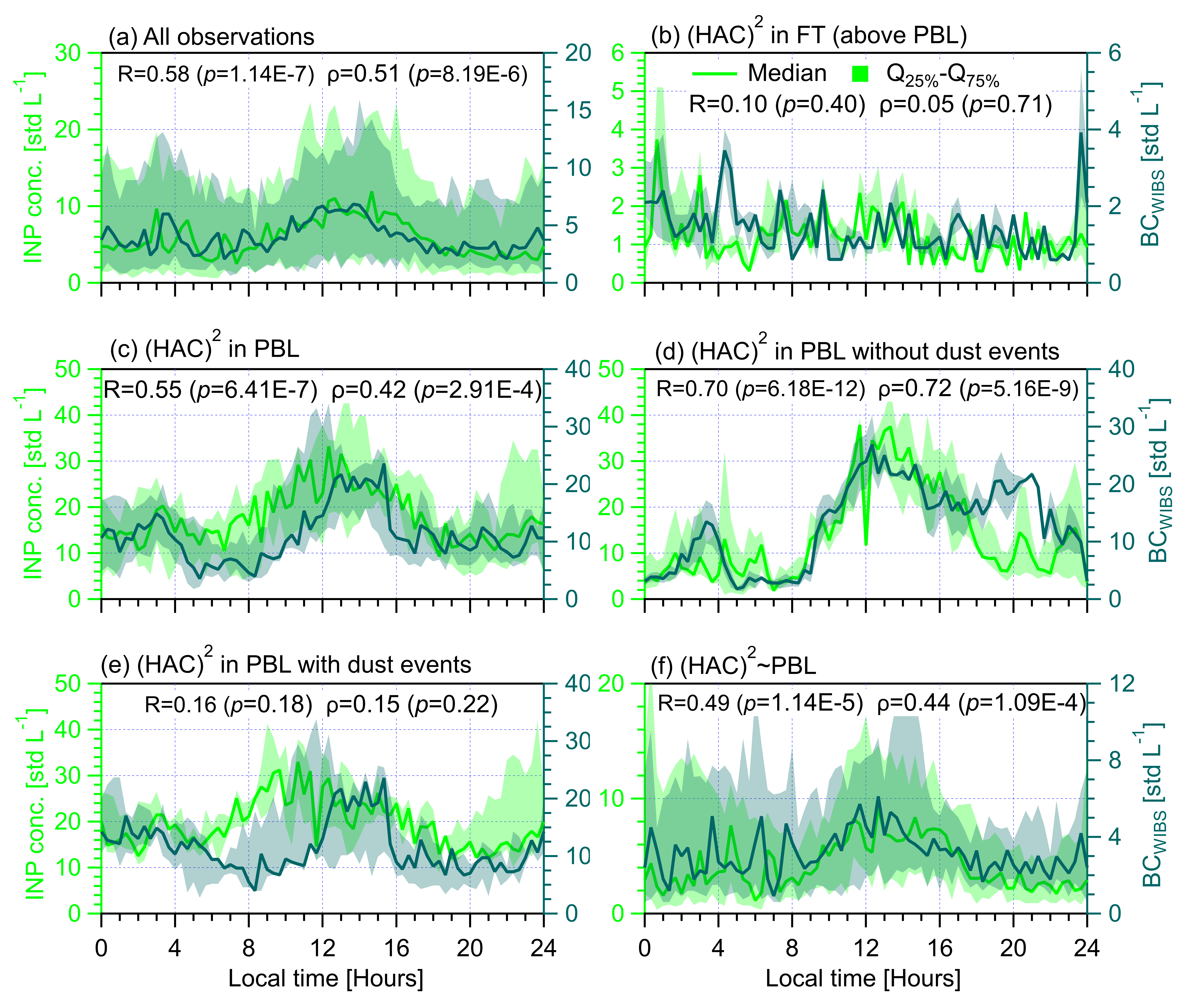


Fig. S27. The same as Fig. S7 but the right axis is for BC_WIBS_.

## S9 The dust event effects on total and fluorescent biological aerosol particles

This section compares the dust event effects on the concentration of total aerosol particles and fluorescent biological aerosol particles (FBAPs). Figure S28 compares the diurnal cycles of total aerosol particles in different size ranges between non-dust and dust days. Figure S29 compares the diurnal cycles of FBAPs in different size ranges between non-dust and dust days.


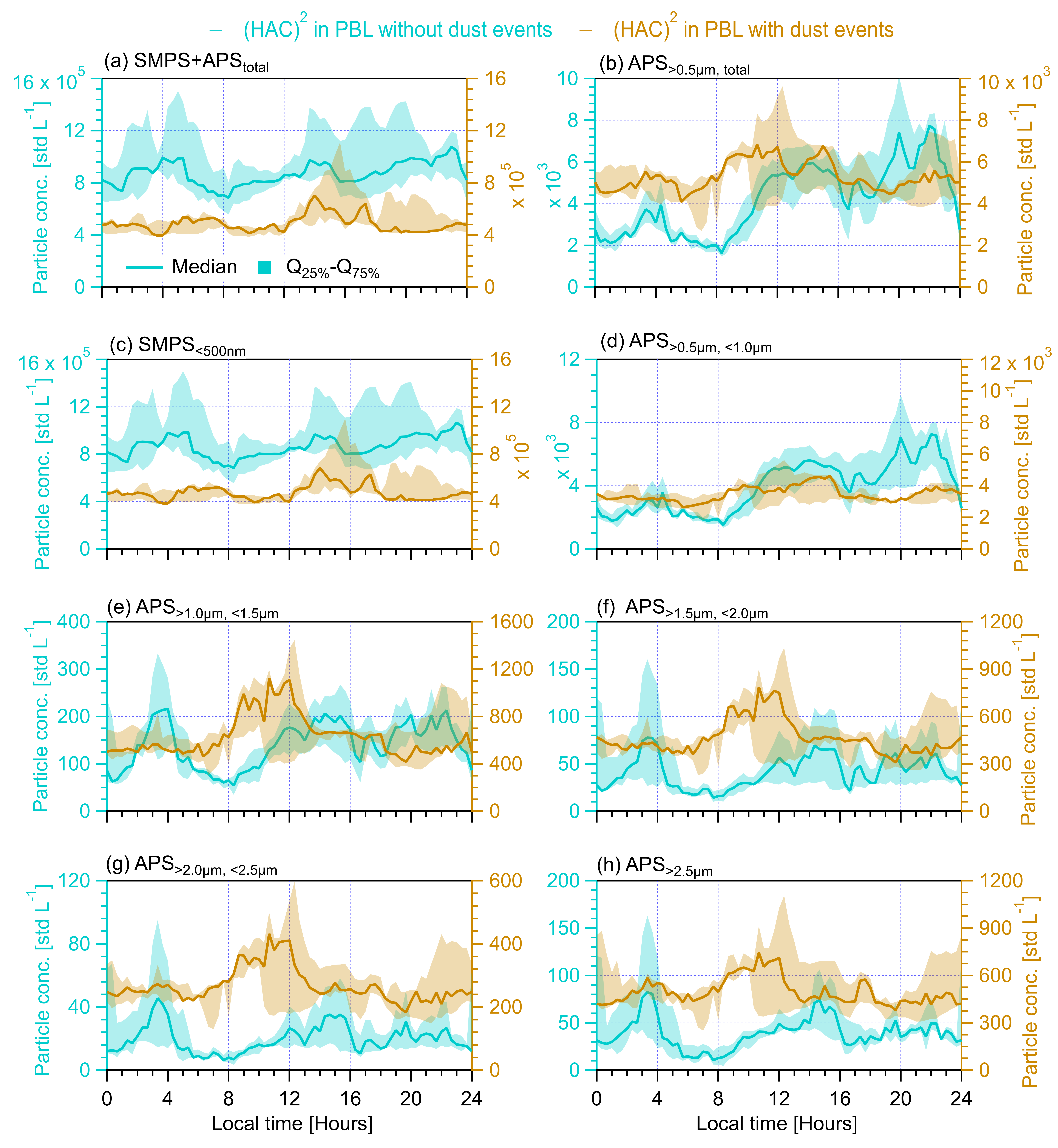


Fig. S 28. Diurnal cycles of total aerosol particles in different size ranges on days without (left axis) and with (right axis) dust events. Solid lines indicate the median value and the shading area around the median line shows the range between 25^th^ and 75^th^ quartiles. (a) SMPS+APS_total_, (b) APS_>0.5μm, total_, (c) SMPS_<500nm_, (d) APS_>0.5μm, <1.0μm_, (e) APS_>1.0μm, <1.5μm_, (f) APS_>1.5μm, <2.0μm_, (g) APS_>2.0μm, <2.5μm_ and (h) APS_>2.5μm_.





Fig. S 29. Diurnal cycles of fluorescent biological aerosol particles in different size ranges on days without (left axis) and with (right axis) dust events. Solid lines indicate the median value and the shading area around the median line shows the range between 25^th^ and 75^th^ quartiles. (a) Fluo_WIBS>0.5μm, total_, (b) Fluo_WIBS>0.5μm, <1.0μm_, (c) Fluo_WIBS>1.0μm, <1.5μm_, (d) Fluo_WIBS>1.5μm, <2.0μm_, (e) Fluo_WIBS>2.0μm, <2.5μm_ and (f) Fluo_WIBS>2.5μm_.

## S10 The distribution of INPs and aerosol properties as a function of PBLH

This section shows additional results about the INP distribution as a function of the PBLH (Fig. S30) and the distributions of different aerosol properties (Fig. S31 to Fig. S36). Figure S30 provides all INP data collected during the campaign, to provide additional results for Fig. 7 in the main text which shows temperature filtered INP data. The different aerosol property distributions as a function of the PBLH helps the understanding of INP distributions in the atmosphere.


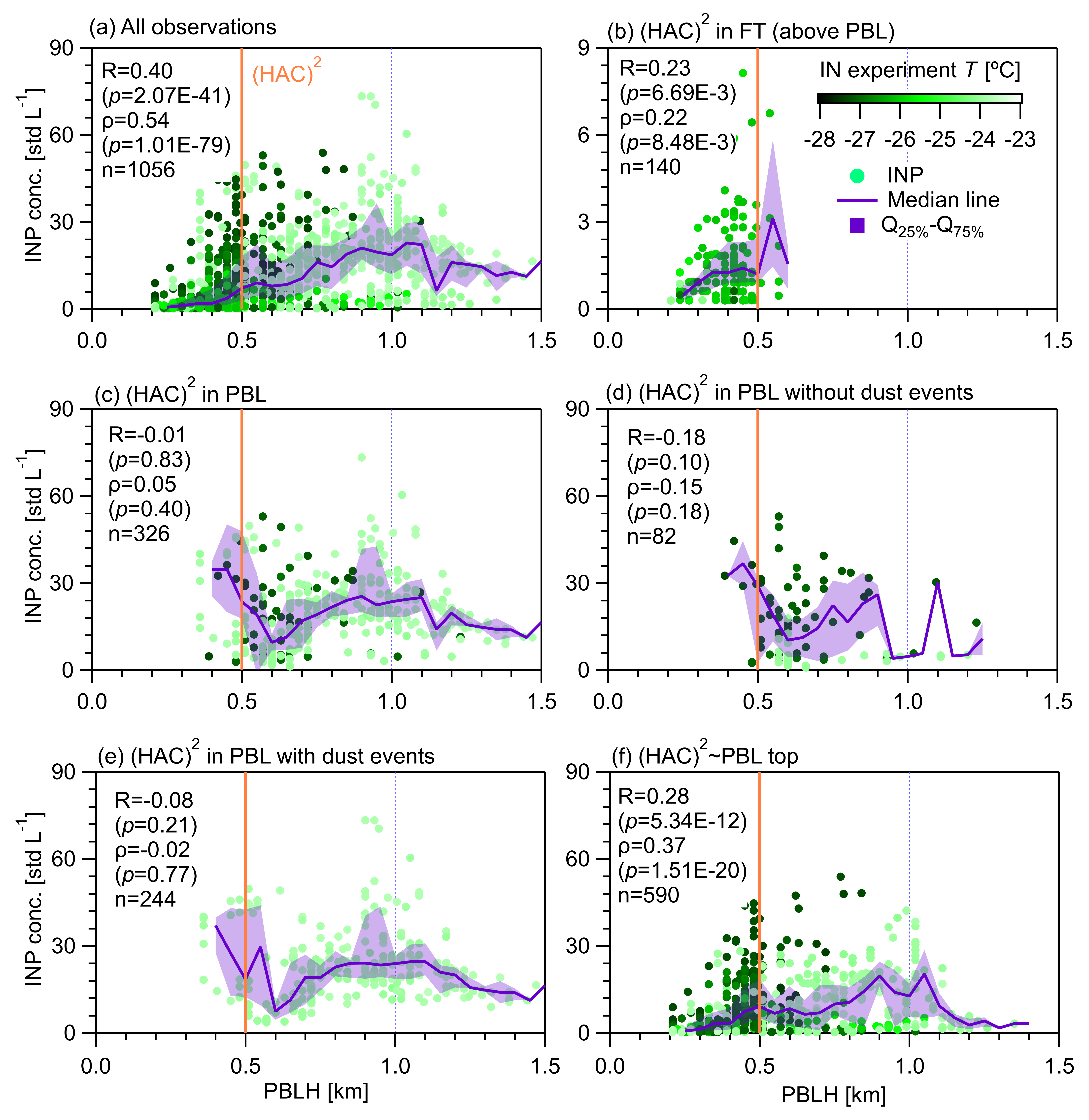


Fig. S30. INP number distribution as a function of PBLH. Solid lines indicate the median value and the shading area around the median line shows the range between 25^th^ and 75^th^ quartiles. Different (HAC)^2^ atmospheric conditions are classified in different panels. (a) All observations during the campaign. (b) For days only in the FT. (c) For days only in the PBL. (d) Days in the PBL without dust events. (e) Days in the PBL with dust events. (f) Observations for days not exclusively in the PBL or FT. Data points are resampled for every 20 min. The Pearson correlation coefficient (*R*) and Spearman’s rank coefficient (ρ), as well as corresponding *p* values, are provided to evaluate the correlation between INP number concentration and PBLH. The *p* value is the probability of obtaining an *R* (ρ) value no smaller than the true *R* (ρ) value if there is no liner correlation between INP and PBLH. The *n* value is the number of data points for the statistical analysis.





Fig. S31. The same as Fig. S30 but the left axis is for SMPS+APS_total_.





Fig. S32. The same as Fig. S30 but the left axis is for APS_>0.5μm, total_.





Fig. S33. The same as Fig. S30 but the left axis is for APS_>2.5μm_.





Fig. S34. The same as Fig. S30 but the left axis is for Fluo_WIBS_.


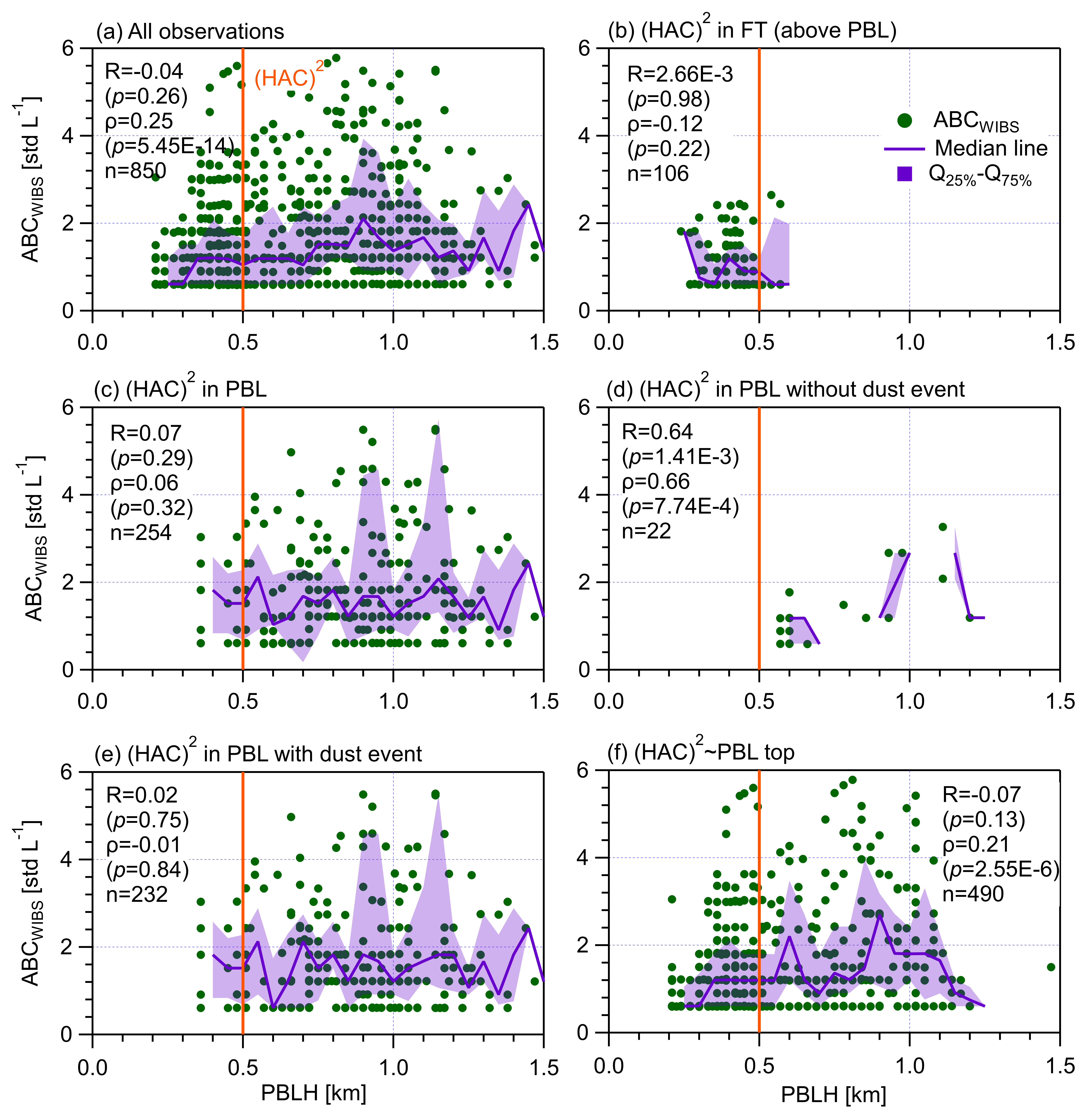


Fig. S35. The same as Fig. S30 but the left axis is for ABC_WIBS_.


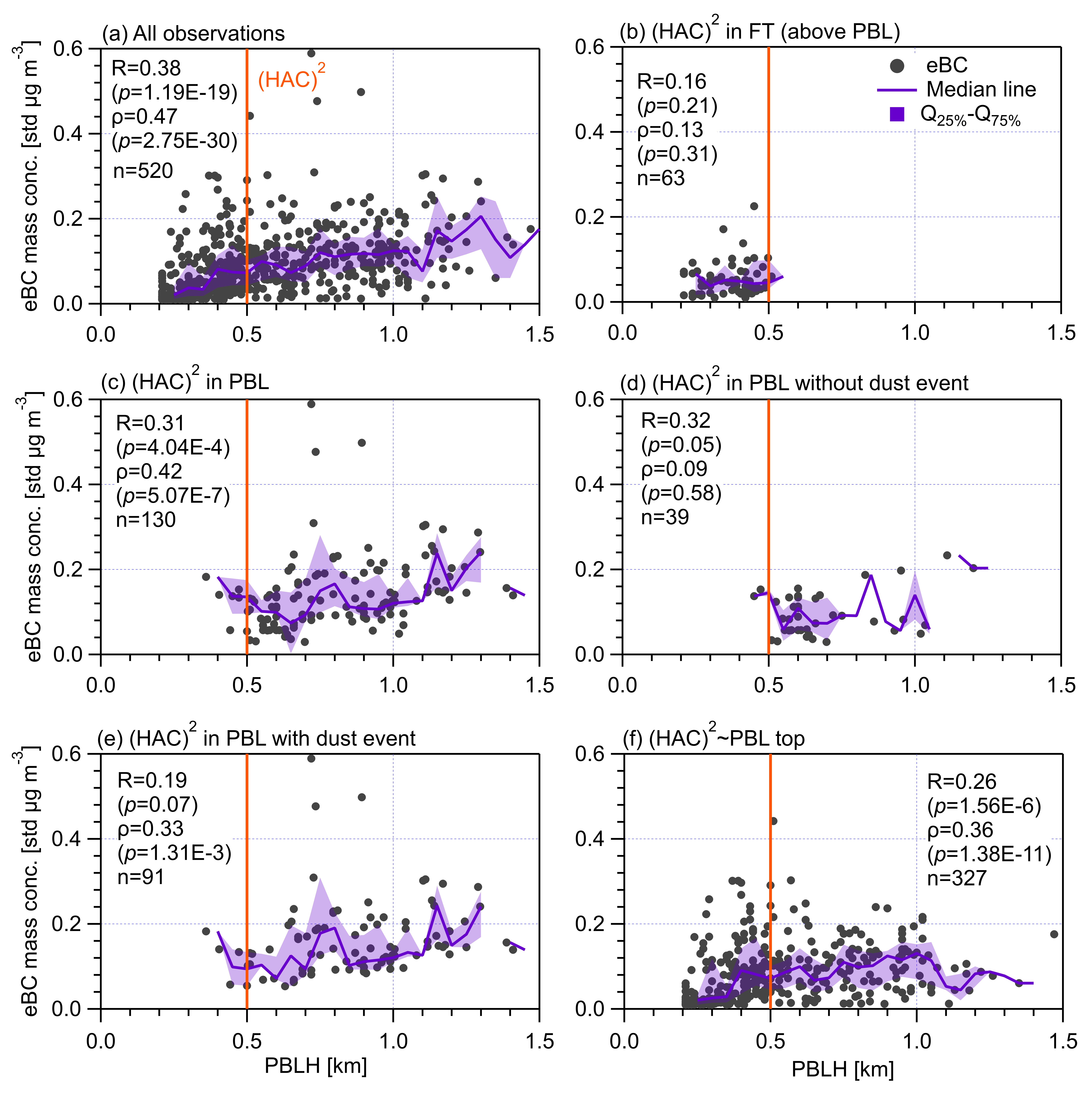


Fig. S36. The same as Fig. S30 but the left axis is for hourly-averaged eBC.

# References

1. Möhler, O., Adams, M., Lacher, L., Vogel, F., Nadolny, J., et al. The Portable Ice Nucleation Experiment (PINE): A New Online Instrument for Laboratory Studies and Automated Long-Term Field Observations of Ice-Nucleating Particles, Atmos. Meas. Tech., **14**, 1143-1166, (2021).

2. Savage, N. J., Krentz, C. E., Könemann, T., Han, T. T., Mainelis, G., et al. Systematic characterization and fluorescence threshold strategies for the wideband integrated bioaerosol sensor (WIBS) using size-resolved biological and interfering particles, Atmos. Meas. Tech., **10**, 4279-4302, (2017).

3. Mordas, G., Prokopciuk, N., Byčenkienė, S., Andriejauskienė, J., and Ulevicius, V. Optical Properties of the Urban Aerosol Particles Obtained from Ground Based Measurements and Satellite-Based Modelling Studies, Adv. Meteorol., **2015**, 1-12, (2015).

4. Krishnamurthy, R., Newsom, R. K., Berg, L. K., Xiao, H., Ma, P.-L., et al. On the estimation of boundary layer heights: a machine learning approach, Atmos. Meas. Tech., **14**, 4403-4424, (2021).

5. Spyrou, C., Mitsakou, C., Kallos, G., Louka, P., and Vlastou, G. An improved limited area model for describing the dust cycle in the atmosphere, J. Geophys. Res., **115**, (2010).

6. Kallos, G., Papadopoulos, A., Katsafados, P., and Nickovic, S. Transatlantic Saharan dust transport: Model simulation and results, J. Geophys. Res. Atmos., **111**, (2006).

7. Khlystov, A., Stanier, C., and Pandis, S. N. An Algorithm for Combining Electrical Mobility and Aerodynamic Size Distributions Data when Measuring Ambient Aerosol Special Issue ofAerosol Science and Technologyon Findings from the Fine Particulate Matter Supersites Program, Aerosol Sci. Technol., **38**, 229-238, (2004).

8. Allabakash, S. and Lim, S. Climatology of Planetary Boundary Layer Height-Controlling Meteorological Parameters Over the Korean Peninsula, Remote Sensing, **12**, (2020).

9. De Wekker, S. F. J. and Kossmann, M. Convective Boundary Layer Heights Over Mountainous Terrain—A Review of Concepts, Frontiers in Earth Science, **3**, (2015).

10. Zardi, D. and Whiteman, C. D.: Diurnal mountain wind systems, Mountain Weather Research and Forecasting: Recent Progress and Current Challenges, NY: Springer, New York, (2013).

11. Asimakopoulos, D. N., Deligiorgi, D. G., and Lalas, D. P. Acoustic Sounder Observations of the Atmospheric Boundary Layer from the Top of a Steep Mountain, Journal of Applied Meteorology and Climatology, **19**, 109-112, (1980).

12. Wieder, J., Mignani, C., Schär, M., Roth, L., Sprenger, M., et al. Unveiling atmospheric transport and mixing mechanisms of ice-nucleating particles over the Alps, Atmos. Chem. Phys., **22**, 3111-3130, (2022).

13. Georgakaki, P., Bougiatioti, A., Wieder, J., Mignani, C., Ramelli, F., et al. On the drivers of droplet variability in alpine mixed-phase clouds, Atmos. Chem. Phys., **21**, 10993-11012, (2021).

14. Collaud Coen, M., Andrews, E., Aliaga, D., Andrade, M., Angelov, H., et al. Identification of topographic features influencing aerosol observations at high altitude stations, Atmos. Chem. Phys., **18**, 12289-12313, (2018).

15. Gini, M., Manousakas, M., Karydas, A. G., and Eleftheriadis, K. Mass size distributions, composition and dose estimates of particulate matter in Saharan dust outbreaks, Environ. Pollut., **298**, 118768, (2022).

16. Petersson Sjögren, M., Alsved, M., Šantl-Temkiv, T., Bjerring Kristensen, T., and Löndahl, J. Measurement report: Atmospheric fluorescent bioaerosol concentrations measured during 18 months in a coniferous forest in the south of Sweden, Atmos. Chem. Phys., **23**, 4977-4992, (2023).
